# Synthetic Routes to, Stabilities and Transformations of, and Characterization of (Carbamoyl)disulfanyl Chlorides and Related Compounds^1^^,^^2^

**DOI:** 10.3390/molecules30193892

**Published:** 2025-09-26

**Authors:** Phillip T. Goldblatt, Tracy R. Thompson, William W. Brennessel, Thomas G. Smith, Alex M. Schrader, Erik S. Goebel, Madeleine J. Henley, Alex Lovstedt, Victor G. Young, George Barany

**Affiliations:** 1 Department of Chemistry, University of Minnesota, Minneapolis, MN 55455, USA; 2 Department of Physical Sciences, Alverno College, Milwaukee, WI 53234, USA; 3 X-Ray Crystallographic Facility, Department of Chemistry, University of Rochester, Rochester, NY 14627, USA

**Keywords:** organosulfur chemistry, (carbamoyl)disulfanyl intermediates and derivatives, 4-methyl-2(3*H*)-benzo-1,2,4-dithiazinone, phosphine sulfurization, X-ray crystallography

## Abstract

Previously unobserved (carbamoyl)disulfanyl chlorides were prepared by (i) addition of limiting aromatic secondary amine to (chlorocarbonyl)disulfanyl chloride; (ii) Harris reactions of sulfur dichloride with appropriate *O*-alkyl *N*-methyl-*N*-arylthiocarbamates; and (iii) regiospecific chlorolysis of bis(*N*-methyl-*N*-arylcarbamoyl)disulfanes. The newly synthesized unstable species were observed *in situ* by ^1^H NMR and were trapped with alkenes, thiocarbamates, and thiols using methods precedented by the chemistry of analogous (carbamoyl)sulfenyl chlorides. Furthermore, each of the trapped products was synthesized by an alternate route, reinforcing conclusions about their structures. While (*N*-methyl-*N*-phenylcarbamoyl)disulfanyl chloride was unstable and decomposed quickly or cyclized intramolecularly, introduction of the *N*,2,6-trimethylphenyl moiety led to significantly improved stability. As part of this study, an interesting, unexpectedly stable 1,2,4-dithiazinone was discovered and its structure was established by X-ray crystallography. The new heterocycle, with its twisted out-of-plane disulfide bond in a six-membered ring, readily donated a sulfur atom to triphenylphosphine; this reaction resulted in the formation of triphenylphosphine sulfide, along with the corresponding highly stable heterocycle in which the single sulfur that remains is part of a planar five-membered ring, fused to a co-planar aryl moiety.

## 1. Introduction

We reported previously [1] on the chemistry of (carbamoyl)sulfenyl chlorides (**1**), species once thought [2] to be unstable, but now recognized to be surprisingly long-lived under ambient conditions. Compounds **1** were characterized by spectroscopic methods and by the elucidation of a number of facile chemical transformations consistent with their assigned parent structures. It then became natural to wonder about the possible existence and properties of (carbamoyl)disulfanyl chlorides (**2**), species that were hinted at briefly in work we carried out in the mid-1980s [3], but for which compelling structural evidence was lacking^3^.

We describe herein how some—albeit not all—of the optimized methods [1] used to generate **1** can be homologated to generate **2** (non-isolable) *in situ*. Furthermore, we report additional unprecedented routes towards **2**, along with direct spectroscopic characterization of **2** (however made) and additional corroborative structural evidence through trapping of **2** with alkenes, thiols, or thiocarbamates. These trapping experiments provided the anticipated products, each of which was stable and accessible by independent routes. Finally, we address the stability of **2**, describe pathways for its decomposition, and announce a novel 1,2,4-dithiazinone heterocycle (**3**), the structure of which was established by X-ray crystallography.

Compound **3**, that features a fused six-membered ring which incorporates an out-of-plane disulfide bond, reacts with triphenylphosphine to form triphenylphosphine sulfide, along with a thiazolone **4** that is well described in the literature [2,3,4,5,6,7,8]. Given that compound **4** has a single sulfur atom within a five-membered ring that is fused to and coplanar with the arene, the fact that **4** is the product when compound **3** is reacted with phosphines reveals a novel driving force for desulfurization reactions. These experimental results and mechanistic insights further suggest that the new dithiazinone **3** has the potential to be a useful sulfurizing agent for DNA and RNA chemistry^4^.

## 2. Results and Discussion

### 2.1. Generation, Stability, and Decomposition Pathways of (Carbamoyl)disulfanyl Chlorides (**2**)

**Scheme 1:**
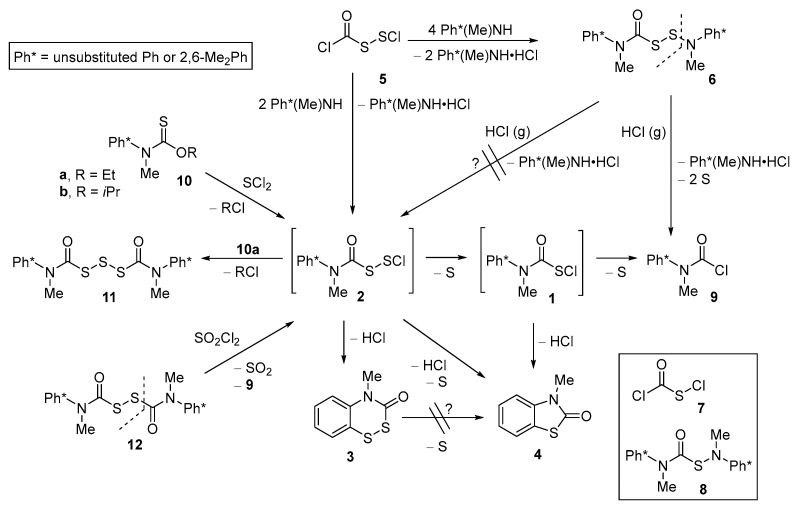
This chemistry was explored for derivatives of unsubstituted *N*-methylaniline [Ph* = Ph; the formula number has no further punctuation] and of *N*,2,6-trimethylaniline [prime = ‘ series; Ph* = 2,6-Me_2_Ph]. As discussed further in the text, (*N*-methyl-*N*-phenylcarbamoyl)disulfanyl chloride (**2**) can be accessed *only* by addition of limiting *N*-methylaniline to (chlorocarbonyl)disulfanyl chloride (**5**) [**2′** can also be prepared in this way]. Transformation of **2** generated *in situ* gives rise to the novel two-sulfur heterocycle **3**, which is stable once formed, but is challenging to obtain pure in good yields because of numerous co-products that are obtained at the same time. Furthermore, most routes aimed at **2** give the one-sulfur heterocycle **4**, sometimes directly, and other times alongside **3**. There is no reason to believe that, under the reaction conditions used, **3** loses a sulfur atom to give **4**. When heterocyclization involving the aromatic ring is blocked, i.e., prime (‘) series with Ph* = 2,6-Me_2_Ph, structures analogous to **3** and **4** are clearly precluded, and additional routes to **2′** become possible. In this regard, **2′** can be accessed by regiospecific chlorolytic cleavage of **12′**, and by treatment of thiocarbamates **10′** with SCl_2_ (1 equiv). However, a potential route to **2** involving treatments of disulfenamides **6** with hydrogen chloride gas was unsuccessful in both the Ph(Me)N and (2,6-Me_2_Ph)(Me)N series.

Initial experiments (Scheme 1) directed at **2** focused on the *N*-methylaniline family [parent series has *regular numbers* which are *not* followed by prime (**‘**)], particularly since our laboratory had available an extensive set of derivatives [1,3,6] that could serve as reference compounds in elucidating the subsequent chemistries.

**Figure 1:**
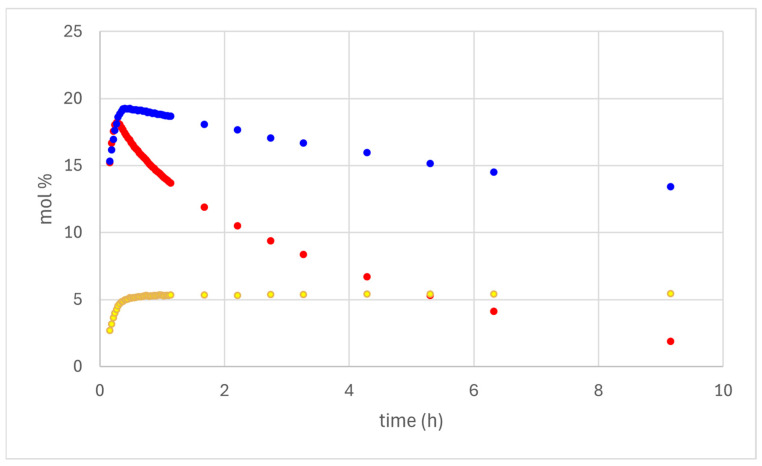
Reaction of *N*-methylaniline (2 equiv) with (chlorocarbonyl)disulfanyl chloride (**5**) at 25 °C in CDCl_3_, as monitored by ^1^H NMR as a function of time. Every discretely measured data point is shown. Reactants were combined at 4 °C and the first time-point was acquired about 8 min thereafter. Given spontaneous exotherms and/or equilibration of temperature to the interior of the NMR probe, it was difficult to discern accurate kinetic parameters from the earliest phases of the reaction. The concentrations once mixing was complete were 0.34 M for **5** and 0.69 M for *N*-methylaniline (ensuring a full second equiv of amine present to neutralize hydrogen chloride formed during the initial acylation), and some experiments included 0.04 M hexamethylbenzene (HMB) as an internal reference standard. Saturation levels of heterocycle **3** (yellow) and **4** (blue) formed fairly quickly; in fact, the apparent amount of **4** decreases due to solubility considerations. With longer times, the amount of intermediate **2** (red) goes to zero, although probably through processes other than conversion to **3**. In other experiments, it was shown (counter-intuitively) that **2**, **3**, and **4** all form even when the final concentration of *N*-methylaniline is equal to, or less than, that of **5** (in all such experiments, the final concentration of **5**, once addition was complete, was always 0.33 M).

To obtain (*N*-methyl-*N*-phenylcarbamoyl)disulfanyl chloride (**2**) *in situ* (Figure 1), limiting^5^^,^^6^ *N*-methylaniline (2 equiv) was added carefully to a solution of (chlorocarbonyl)disulfanyl chloride (**5**) [3,14,15,16,17,18] (1 equiv) in CDCl_3_, whereupon **2** was recognized by a diagnostic ^1^H NMR apparent singlet^7^ at δ 3.66. Analogous to the fact that the corresponding one-sulfur (carbamoyl)sulfenyl chloride **1** (diagnostic singlet at δ 3.38) [1] gradually cyclizes to form 3-methyl-2(3*H*)-benzothiazolone (**4**) [2,3,4,5,6,7,8] (diagnostic singlet at δ 3.45), (carbamoyl)disulfanyl chloride **2** was found to have limited stability and cyclizes to 4-methyl-2(3*H*)-benzo-1,2,4-dithiazinone (**3**) [diagnostic singlet at δ 3.52; see later for more about this *novel* heterocycle] relatively quickly [t_½_~8 min at 25 °C in CDCl_3_; rate independent^8^ of concentration of **2**].

The heterocyclization of (carbamoyl)disulfanyl chloride **2** to provide compound **3** took place concurrent to the desulfurization of compound **2** to (carbamoyl)sulfenyl chloride **1**, a process that could be tracked directly by ^1^H NMR (Figure 1), and indirectly by trapping experiments (see next section) carried out as a function of time. Moreover, as **1** formed, it too decomposed further in the previously documented manner [1], resulting in the accumulation of varying quantities of heterocycle **4** and carbamoyl chloride **9 [3,6]** over the course of several h at 25 °C. Finally, the act of concentrating [*in vacuo* or under a stream of N_2_] any solution of (carbamoyl)disulfanyl chloride **2**, even shortly after this compound had been freshly generated, resulted in complete decomposition of **2**. Instead, heterocycles **3** and **4** were observed, along with carbamoyl chloride **9**, (carbamoyl)disulfenamide **6**, and *N*-methylaniline hydrochloride [typical ratios~1:3:1:1:6].

In contrast to the successful method just reported, other chemistries [1] that had been developed to generate **1** did *not* homologate well when directed at **2**. For example, hydrogen chloride gas was bubbled through a CDCl_3_ solution of (carbamoyl)disulfenamide **6**—these are conditions expected to specifically cleave the S–N bond^9^ and thereby furnish **2**. While starting **6** was clearly transformed, as evidenced by production of a full equiv of *N*-methylaniline hydrochloride, only trace **2** was noted (< 5%). Moreover, there was no appreciable level of compound **3**, which would have corresponded to heterocyclization of **2**. Instead, a mixture of products was observed, with the major component being the (sulfur-free) carbamoyl chloride **9** [not isolated, but unambiguously noted in the ^1^H NMR spectrum].

In a different approach^10^^,^^11^, a CDCl_3_ solution of thiocarbamate **10b** was treated with sulfur dichloride (SCl_2_, 1 equiv). Within 10 min at 25 °C, starting material had been entirely consumed, and *i*PrCl (1 equiv) had formed, along with heterocycle **4** (~65%). Neither **2** nor **3** was observed at any point in this reaction, but the presence of bis(*N*-methyl-*N*-phenylcarbamoyl)trisulfane (**11**) (~35%, accounted for by a Harris reaction [20] of **2** with **10b**) suggested that **2** must have been present—even if only as a relatively short-lived intermediate.

Lastly, as has already been reported [1], regiospecific chlorolysis of disulfane **12**, using SO_2_Cl_2_ as the cleavage reagent, breaks the acyl sulfur bond rather than the sulfur–sulfur bond, meaning that compound **2**, along with carbamoyl chloride **9**, could be anticipated. While compound **9** (quantitative, based on **12**) was indeed observed, any transient **2** that might have formed rapidly decomposed under these reaction conditions *not* to **3**, but rather *directly* to **4** (with elemental sulfur presumably formed as a co-product).

All reactions designed to access (carbamoyl)disulfanyl chlorides (**2** in Scheme 1) were repeated in the *N*,2,6-trimethylaniline family (prime = ‘ series), based on the rationale that the two methyl groups on the aromatic ring might render desired product **2′** more stable and/or promote alternate decomposition pathways, since any putative intramolecular heterocyclizative side reactions would no longer be possible. Thus, it was a pleasant surprise to find that not only was **2′** obtained by reaction of limiting *N*,2,6-trimethylaniline with (chlorocarbonyl)disulfanyl chloride (**5**), but also that regiospecific chlorolytic cleavage (t_½_~4 h) of disulfane **12′** cleanly gave desired **2′** plus carbamoyl chloride **9′** [1,21], in a 1:1 ratio^12^. Most importantly, Harris reaction [20] of thiocarbamate **10a’** with SCl_2_ (1 equiv) gave **2′** cleanly, along with EtCl (1 equiv).

After more than a week at 25 °C, compound **2′** eventually lost two sulfurs to give additional levels of **9′**. It seems reasonable to speculate that a decomposition pathway of **2′** (two sulfurs) to **1′** (loss of one sulfur) to **9′** (sulfur-free carbamoyl chloride) takes place, but there is no definitive evidence that this is the case.

Earlier, we mentioned the complete instability of **2** upon concentration of a CDCl_3_ solution that contained the species **2** generated *in situ*. In contrast, the extent of decomposition of **2′**, generated in CDCl_3_ solution by any of a variety of methods (Scheme 1), depended on the mode of concentration. Evaporation *in vacuo* or by any process involving heating above 25 °C resulted in complete conversion to carbamoyl chloride **9′** plus two equiv of elemental sulfur. However, when the CDCl_3_ was removed under a gentle stream of N_2_, as much as 70% of **2′** survived.

### 2.2. Trapping of (Carbamoyl)disulfanyl Chlorides (**2**)

**Scheme 2:**
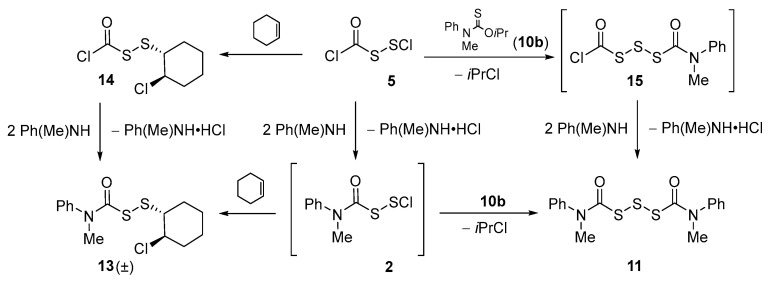
Representative reactions of (carbamoyl)disulfanyl chloride **2**. The chemistry in the scheme was demonstrated with derivatives of unsubstituted *N*-methylaniline [Ph* = Ph]. The stereochemistry of **13** and **14**, drawn as *trans* to correspond to *anti* addition of the corresponding disulfanyl chlorides to the alkene, was not proven explicitly, but several literature precedents [1,22] support such an assignment.

(*N*-Methyl-*N*-phenylcarbamoyl)disulfanyl chloride (**2**), generated by reaction of limiting secondary amine with **5**, was trapped successfully by a number of precedented techniques [1,2,3,13] to give the expected adducts, some new and others previously known. For example, trapping of **2** with cyclohexene gave the novel 2-chlorocyclohexyl(*N*-methyl-*N*-phenylcarbamoyl)disulfane (**13**) (racemic mixture), in a ratio of ~1:1 with the corresponding known sulfide [1], and reaction of **2** with thiocarbamate **10b** (1 equiv) gave bis(*N*-methyl-*N*-phenylcarbamoyl)trisulfane (**11**) [3] (see also footnote 10). In each case, the *same* end products could be obtained by *first* treating (chlorocarbonyl)disulfanyl chloride (**5**) with either cyclohexene or the thiocarbamate to provide relatively unstable derivatives [disulfane **14** and trisulfane **15**, respectively], which were then quenched with *N*-methylaniline (Scheme 2).

**Scheme 3:**
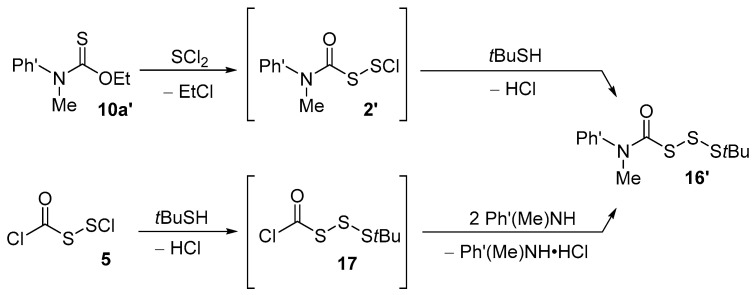
Representative reactions of (carbamoyl)disulfanyl chloride **2′**. The chemistry in the scheme was carried out *specifically* for Ph* = Ph’ = 2,6-Me_2_Ph (the prime series).

Additional chemistry was demonstrated (Scheme 3) on (*N*,2,6-trimethyl-*N*-phenylcarbamoyl)disulfanyl chloride (**2′**), a substrate that was accessible by multiple routes, proved to be far more stable than unsubstituted **2**, and was easier to characterize spectroscopically. In one example, 2-methyl-2-propanethiol was added to freshly prepared **2′** to form *tert*-butyl (*N*,2,6-trimethyl-*N*-phenylcarbamoyl)trisulfane (**16′**) (top of Scheme 3). Alternatively, (chlorocarbonyl)disulfanyl chloride (**5**) could be reacted with the thiol first, providing **17** as an intermediate *in situ*, and subsequent *N*-acylation gave the identical final product **16′** (bottom of Scheme 3).

### 2.3. Reaction of 4-Methyl-2(3H)-benzo-1,2,4-dithiazinone (**3**) with Triphenylphosphine

**Scheme 4:**
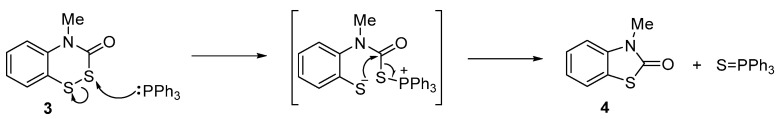
Reaction of 4-methyl-2(3*H*)-benzo-1,2,4-dithiazinone (**3**) with triphenylphosphine to cleanly provide 3-methyl-2(3*H*)-benzothiazolone (**4**), together with triphenylphosphine sulfide. The species shown in the middle of the Scheme is a plausible intermediate for this transformation, and the arrows indicating movements of electrons are suggestive of the likely mechanism, including identification of the “active” sulfur atom which happens to be the out-of-plane sulfur in the X-ray structure of **3** (see Section 2.4). Compare this proposed mechanism to the elegant work of Hanusek *et al*. [23,24,25], who elucidated mechanisms for the desulfurization of EDITH and related compounds with P(III) reagents.

This research provided access for the first time to 4-methyl-2(3*H*)-benzo-1,2,4-dithiazinone (**3**), a novel heterocycle that was generated by slow addition of *N*-methylaniline to (chlorocarbonyl)disulfanyl chloride (**5**), and then purified by flash chromatography. For reasons already touched upon in the Introduction, we wondered whether **3** could donate a sulfur atom to trivalent phosphorus, and chose triphenylphosphine as a model substrate. Experiments (Scheme 4) were carried out with **3** in excess over Ph_3_P, or *vice versa*. In each case, reactions in CDCl_3_ solution at 25 °C were exceptionally rapid, with whatever the limiting compound had been entirely absent upon the earliest possible ^1^H or ^31^P NMR examination. Importantly, when excess **3** was used, negligible levels (< 2%) of triphenylphosphine oxide were observed as a side reaction to the main process (Scheme 4).

### 2.4. Crystal Structure of 4-Methyl-2(3H)-benzo-1,2,4-dithiazinone (**3**) and Comparison to Linear and Cyclic Compounds with One or Two Sulfur Atoms

Material suitable for X-ray crystallographic analysis was obtained by dissolving purified **3** in suitable mixtures of halogenated hydrocarbon (i.e., CH_2_Cl_2_ or CDCl_3_) and nonpolar hydrocarbon (i.e., hexane or heptane), followed by slow partial evaporation.

**Figure 2:**
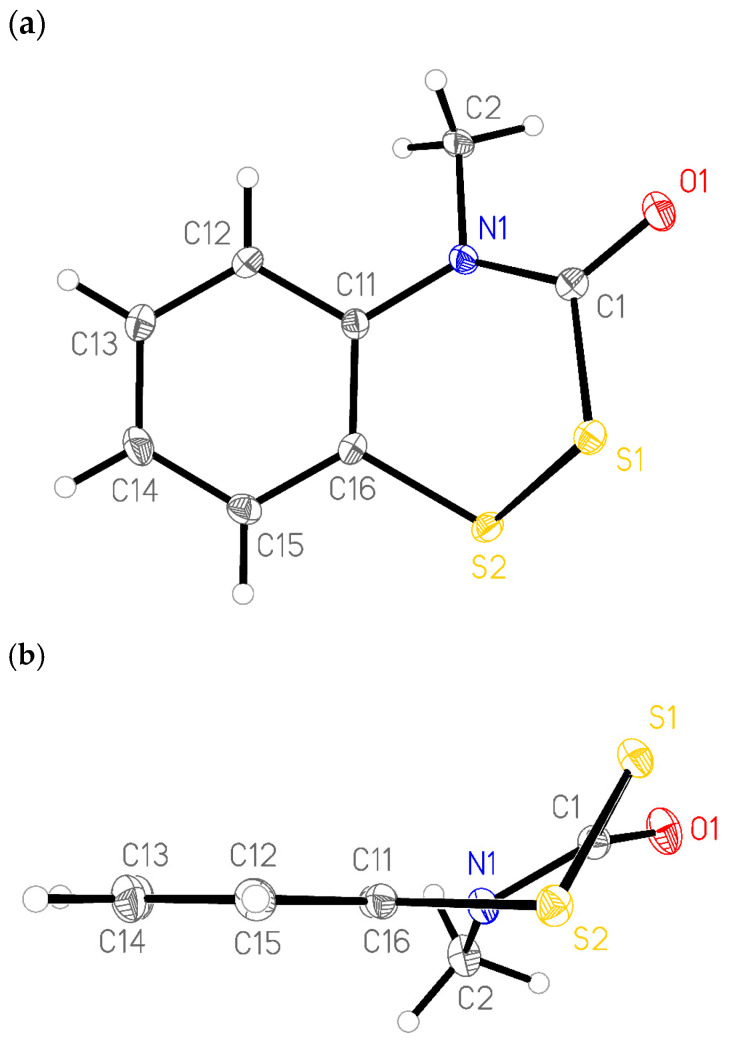
(**a**) Anisotropic displacement ellipsoid plot of dithiazinone **3**, drawn at the 50% probability level. All non-H atoms are labeled and numbered. The crystallographic numbering scheme differs from IUPAC numbering. (**b**) On-edge view showing the deviation from planarity.

Figure 2 shows the solved structure of **3**, while Table 1 lists select bond lengths, bond angles, and torsion angles. The six atoms of the heterocyclic ring of **3** are notably *not* coplanar, in stark contrast to the coplanarity of the five atoms (fused to the benzene ring) of heterocycle **4** [7,8], which has only a single sulfur atom and is essentially planar [RMS deviation from planarity is 0.008]. The heterocyclic ring in **3** has two bond angles, C1–S1–S2 and C16–S2–S1, that are less than 100°, suggesting considerable angle strain, and has a 0.328 Å deviation from planarity.

**Scheme 5:**
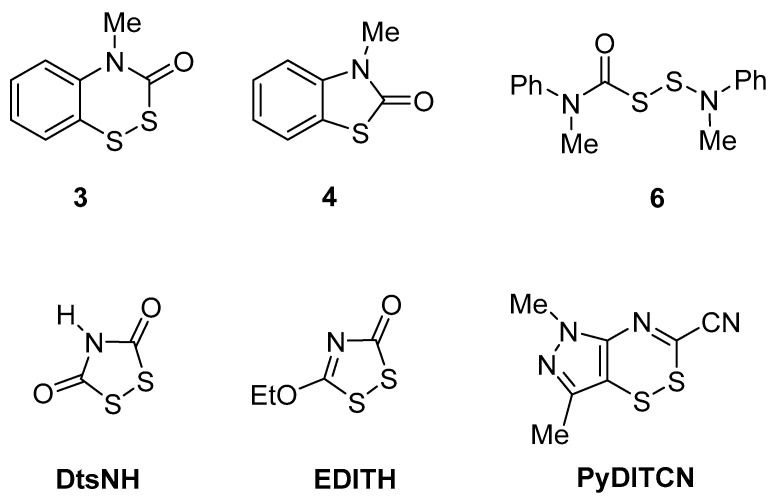
Chemical structures of **3** and several comparison compounds (Table 1).

Compound **3** was compared to somewhat related compounds previously reported in the literature (Table 1, Scheme 5). The dihedral (torsion) angle of the C–S–S–C (C1–S1–S2–C16) group in **3** is −58.36(8)°, in contrast with the near-zero values of the corresponding dihedral angle seen in planar five-membered heterocyclic disulfides like 3-ethoxy-1,2,4-dithiazolin-5-one (EDITH) [26,27] and 1,2,4-dithiazolidine-3,5-dione (DtsNH) [26], and also in contrast with the preferred disulfide dihedral angle of 90° for acyclic disulfides, including those found in simple compounds [28,29], in cystine alone [30], or in cystine residues that are incorporated into peptides and proteins [31]. On point, a C–S–S–N torsion angle of −92.62(6)° is found in (*N*-methyl-*N*-phenylamino)(*N*-methyl-*N*-phenylcarbamoyl)disulfane (**6**) [13], which can be thought of as a linearized analogue of **3**. On the other hand, the S–S bond length in heterocyclic **3**, namely 2.0580(6) Å, is quite close to the corresponding value for linear **6**, namely 2.0625(5) Å.

**Table 1:** Comparison of selected bond lengths (Å), angles (°), and torsion angles (°) of compound **3** with several other compounds, drawn in Scheme 5, that have related structural features.

Name or Number CSD Refcode [32] CCDC Number	3 2470096	PyDITCN SUJQAR 1013809	6 VUYBIC 1428652	4 COSFAR01 711838	EDITH 2479242	DtsNH NAHMUE 123954
S1–C1	1.7976 (18)	1.784 (3)	1.8273 (13)	1.776 (2)	1.806 (7)	1.764 (2)
S1–S2	2.0580 (6)	2.0655 (9)	2.0625 (5)	NR	2.051 (2)	2.0584 (8)
S2–C16	1.7627 (18)	1.754 (3)	1.6660 (11) ^a^	1.744 (2) ^d^	1.757 (7)	1.761 (2)
N1–C1	1.363 (2)	1.287 (3)	1.3569 (16)	1.367 (2)	1.385 (10)	1.367 (2)
N1–C11	1.427 (2)	1.375 (3)	1.4429 (15)	1.392 (2)	1.272 (9)	1.369 (2)
C11–C16	1.403 (2)	1.389 (4)	1.3865 (18)	1.392 (3)	NR	NR
C1–S1–S2	98.78 (6)	98.13 (9)	102.60 (4)	NR	94.5 (3)	95.42 (6)
C16–S2–S1	97.43 (6)	95.67 (9)	108.37 (4) ^b^	NR	91.6 (2)	95.45 (6)
C1–N1–C11	126.11 (15)	117.2 (2)	123.17 (10)	115.4 (2)	115.1 (6)	121.99 (14)
N1–C1–S1	117.55 (13)	126.9 (2)	112.55 (9)	109.17 (15)	115.3 (5)	113.51 (12)
C16–C11–N1	122.13 (15)	131.2 (2)	NR	113.2 (2)	NR	NR
C11–C16–S2	118.89 (13)	120.3 (2)	NR	110.42 (14) ^d^	NR	NR
C1–S1–S2–C16	–58.36 (8)	51.72 (12)	–92.62 (6) ^c^	NR	1.6 (4)	–1.94 (7)
C2–N1–C1–O1	0.5 (3)	NR	4.27 (19)	1.1 (2)	NR	NR
N1–C11–C16–S2	0.8 (2)	0.2 (4)	NR	0.21 (14) ^d^	NR	NR
Ring deviation from planarity	0.328	0.292	NR	0.008	0.011	0.013

Compound **3** is from this work, with values provided in this table excerpted from Tables S4 and S6; one-sulfur compound **4** was previously solved [7,8] and we are using data from [8]; the linear (carbamoyl)disulfenamide **6** was reported in [13]; essentially planar five-membered cyclic dithiazolines EDITH and DtsNH are originally from [26] and we are using data from [27] for EDITH; six-membered dithiazine PyDITCN, which is fused to an aromatic pyrazole, is from [33]. Atom numbers for each reference compound were adjusted to align with compound **3**, as defined in Figure 2. NR means “not relevant”. Notes: (a) for compound **6** this is S–N; (b) for compound **6** this is S–S–N; (c) for compound **6** this is C–S–S–N; (d) for compound **4**, there is only one S.

A search of the Cambridge database uncovered over fifty six-membered rings each with an S–S–C–N–C–C composition (in that order). All but one of these includes sp^3^ carbon atoms, and many of them are bicyclic piperazinediones. In this group, the average C–S–S–C torsion angle is ~12°, with a range of values from 2° through 20° (e.g., [34,35,36,37,38]). Thus, inclusion in a ring leads to dihedrals close to 0°, although not necessarily exactly 0° as is the case for DtsNH and EDITH for reasons covered in the next paragraph.

**Figure 3:**
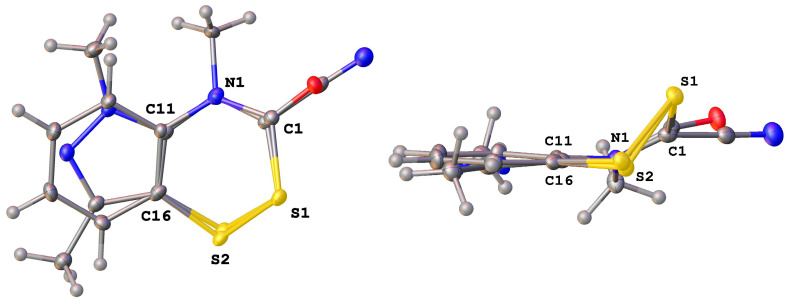
Overlay of 4-methyl-2(3*H*)-benzo-1,2,4-dithiazinone (**3**) with closely related 1,3-dimethyl-5*H*-pyrazolo [3,4-*e*][1,2,4]dithiazine-3-carbonitrile (PyDITCN). To demonstrate the overlay, atoms S1, N1, and C16 from the two molecules were restrained to have similar positions, and two vantage points are shown.

Only one structure, namely 1,3-dimethyl-5*H*-pyrazolo[3,4-*e*][1,2,4]dithiazine-3-carbonitrile (PyDITCN) [33], was found to be extraordinarily similar to **3** (Figure 3). Both compounds feature a six-atom heterocyclic C–N–C–S–S–C group fused to an aromatic ring (benzene for **3**; 1*H*-pyrazole for PyDITCN). In both cases (**3** and PyDITCN), the N and C atoms in the dithiazine ring are sp^2^ hybridized, thus differentiating them from other compounds which have sp^3^ hybridized carbon atoms bonded to S (see previous paragraph). The adjacent aromatic ring causes the disulfide-containing heterocycle to deviate from planarity (0.292 Å in PyDITCN; 0.328 Å in **3**), and forces significant pucker of a sulfur atom to preclude the formation of an antiaromatic ring with eight π electrons. In contrast, the comparison five-membered heterocyclic disulfides DtsNH and EDITH have six π electrons, and the aromatic stabilization from planarity takes priority over the maximally unfavorable disulfide dihedral of 0°.

The structural information about these compounds has interesting practical implications. We hypothesize that the ring strain introduced by the extra sulfur in **3** and/or a strong driving force to gain planarity, might help to explain the rapid phosphine-induced desulfurization of **3** to **4** (Scheme 4 and footnote 4). Furthermore, we predict that PyDITCN, the compound reported by Koutentis [33] which is similar to **3** by being a fused thiazine with an out-of-plane sulfur atom (see previous paragraph), could be a potent sulfurizing agent as well.

## 3. Materials and Methods

### 3.1. General

For the most part, we used starting materials and reagents, and followed protocols, that were described in our previous publications [1,3,6,18]. Ordinary laboratory glassware and equipment were used, with precautions such as maintenance of a dry or oxygen-free atmosphere, for example with Schlenk lines, proving to be entirely unnecessary. Sulfur dichloride (SCl_2_) was purified by distillation from PCl_3_, at atmospheric pressure, and stored cold over PCl_5_. When (carbamoyl)disulfanyl chlorides **2** or **2′** were generated with the intention being to probe their further transformations, the follow-up reactions were conducted within 5 min. (Chlorocarbonyl)disulfanyl chloride (**5**) was prepared following Schroll and Barany [3], as elaborated further in Hammer *et al*. [18]; in brief, SO_2_Cl_2_ (4.8 equiv) was added to isopropyl xanthic anhydride (1 equiv), and desired **5** was obtained directly upon “cracking” vacuum distillation, bp 29–41 °C (0.47–0.54 mm). This procedure was robust, carried out frequently in our Minneapolis laboratories, and reproduced relatively recently by Chemveda Life Sciences (Hyderabad, India). *N*,2,6-trimethylaniline (TMA), made in-house for most of our work (as per [1,3]), was recently obtained commercially from AmBeed (Arlington Heights, IL, USA).

Some compounds were purified by silica gel flash column chromatography, using either standard laboratory glassware and supplies, or using a Biotage Isolera Prime instrument with Silicycle Flash cartridges (40–63 µm, 60-Å silica gel) (Biotage, Uppsala, Sweden).

Separations and accurate mass measurements (HRMS) were carried out on a Sciex Exion UHPLC coupled to a Sciex X500R quadrupole time-of-flight (qtof) mass spectrometer (SCIEX, Framingham, MA, USA). An Agilent XDB-C_18_ 2.1 mm × 100 mm column (1.7 μm particles) at 40 °C was used during the following 14 min gradient separation with 0.1% aqueous formic acid (A) and 0.1% methanolic formic acid (B), at a flow rate of 0.5 mL/min: 10% B, 0–2 min; 10% B to 80% B, 2–7 min; 80% B 7–8 min; 80% B to 97% B, 8–9 min; 97% B, 9–11 min; 97% B to 10% B, 11–12 min. Electrospray ionization mass spectra in positive ionization mode were collected over the range *m*/*z* 50–1200 during the analysis. MS parameters were as follows: ion source gas 1: 38 psi; ion source gas 2: 38 psi; curtain gas: 30 psi; CAD gas: 7; temperature: 500 °C; spray voltage: 5500 V; declustering potential: 50 V; DP spread: 0 V; CE: 10 V; CE spread: 0 V.

The elemental analysis for **6′** was obtained at the CENTC Elemental Analysis Facility at the University of Rochester. Microanalysis samples were weighed with a PerkinElmer Model AD6000 Autobalance (PerkinElmer, Shelton, CT, USA) and their compositions were determined with a PerkinElmer 2400 Series II Analyzer (PerkinElmer, Shelton, CT, USA).

NMR data were acquired with CDCl_3_ as solvent at 25 °C on Varian (Varian, Inc., Palo Alto, CA, USA), Bruker (Bruker Corporation, Billerica, MA, USA) or JEOL (JEOL USA, Inc., Peabody, MA, USA) spectrometers with 5 mm probes, operating at ^1^H frequencies of 300, 400, or 500 MHz, ^13^C frequencies of 75, 101, or 126 MHz, and a ^31^P frequency of 162 MHz. All ^1^H, ^13^C, and ^31^P chemical shifts were referenced, directly or indirectly, to an internal standard, tetramethylsilane (TMS, δ 0 ppm), or to the solvent (δ 7.26 for ^1^H and δ 77.0 for ^13^C), following IUPAC recommendations [39,40].

NMR data were processed using MestReNova version 16.0.0-39276 (Mestrelab Research S.L.U., Santiago de Compostela, Spain); this included the capability to analyze kinetic studies (e.g., Figure 1).

X-ray data collection and structure solution were conducted at the X-ray Crystallographic Laboratory, Department of Chemistry, University of Minnesota. X-ray data collection and structure refinement details are given below and in the Supplemental Information (Tables S1–S7).

### 3.2. X-Ray Data Collection, Solution, and Refinement

The description that follows refers to analysis conducted in 2025 with state-of-the-art equipment, which corroborated and improved upon an earlier analysis conducted in 2015 with older equipment. A crystal (approximate dimensions 0.250 × 0.170 × 0.100 mm^3^) was placed onto the tip of a 0.15 mm MiTeGen loop and mounted on a Bruker-AXS D8 Venture diffractometer (Madison, WI, USA) for a data collection at 100(2) K [41]. A preliminary set of cell constants was calculated from reflections harvested from three sets of frames. These initial sets of frames were oriented such that orthogonal wedges of reciprocal space were surveyed. This produced initial orientation matrices determined from 347 reflections. The data collection was carried out using MoK*α* radiation (graphite monochromator) with a frame time of 5.0 s and a detector distance of 5.0 cm. A strategy program was used to assure complete coverage of all unique data to a resolution of 0.75 Å. All major sections of frames were collected with 1.2° steps in *ω* or *φ* at different detector positions in 2*θ*. The intensity data were corrected for absorption and decay (*SADABS*) [42]. Final cell constants were calculated from the *xyz* centroids of 9975 strong reflections from the actual data collection after integration (*SAINT*) [43]. Please refer to Table S1 for additional crystal and refinement information.

The structure was solved using *SHELXT* [44] and refined using *SHELXL* [45]. The space group *Cc* was determined based on systematic absences and intensity statistics. A direct-methods solution was calculated which provided most non-hydrogen atoms from the E-map. Full-matrix least squares/difference Fourier cycles were performed which located the remaining non-hydrogen atoms. All non-hydrogen atoms were refined with anisotropic displacement parameters. All hydrogen atoms were placed in ideal positions and refined as riding atoms with relative isotropic displacement parameters. The final full matrix least squares refinement converged to *R*1 = 0.0181 and *wR*2 = 0.0484 (*F*^2^, all data).

### 3.3. Experimental

**(*N*-Methyl-*N*-phenylcarbamoyl)disulfanyl chloride (2).** A solution of *N*-methylaniline (73 mg, 0.70 mmol) in CDCl_3_ (0.7 mL) was added dropwise, over 5 min, to a solution of (chlorocarbonyl)disulfanyl chloride (**5**) (58 mg, 0.35 mmol) in CDCl_3_ (0.35 mL), at 4 °C. ^1^H NMR spectroscopic examination within 10 min revealed the title product [diagnostic singlet at δ 3.66] along with significant amounts of heterocycles **3** and **4** [diagnostic singlets at δ 3.52 and 3.45 respectively] and trace amounts of (carbamoyl)disulfenamide **6** [diagnostic singlets at δ 3.35 and 3.38]. After 8 h, neither **2** nor **1** [diagnostic singlet at δ 3.37] were observed. A quantitative amount of *N*-methylaniline hydrochloride (1 equiv) was observed at all timepoints. More information about this reaction is found in Figure 1 and its legend, as well as the accompanying text.

**(*N*,2,6-Trimethylphenylcarbamoyl)disulfanyl chloride (2′)**. **Method A.** A solution of bis(carbamoyl)disulfane **12′** [1] (155 mg, 0.4 mmol) and SO_2_Cl_2_ (42 μL, 0.52 mmol) in CDCl_3_ (2.0 mL) was examined by ^1^H NMR at 25 °C. Within minutes of combining the reactants, title product **2′** plus carbamoyl chloride **9′** were observed to appear in a 1:1 ratio; this ratio was unchanged throughout the time course of the reaction [t_1/2_ ~4 h for transformation of **12′** to **2′** plus **9′**], and was also found once the end point was reached [starting **12′** entirely consumed after 2 days]. The spectra were deconvoluted to determine the following peaks due to title **2′** in the reaction mixture: δ 7.2 (m, 1H), 7.1 (m, 2H), 3.32 (s, 3H), 2.24 (s, 6H), along with the following peaks attributed to co-product **9′**: δ 7.1–7.2 (m, 3H), 3.25 (s, 3H), 2.27 (s, 6H) [1]. Similarly, the ^13^C spectrum provides evidence for **2′** [δ 162.1, 137.6, 129.2, 129.0, 37.4, 17.5] and **9′** [149.6, 135.5, 128.8, 128.6, 36.6, 17.5]. For reference, the starting **12′** had the following ^1^H spectrum: δ 7.2–7.1 (m, 6H), 3.32 (s, 3H), 3.27 (s, 3H), 2.32 (s, 6H), 2.24 (s, 6H). **Method B.** A solution of *N*,2,6-trimethylaniline (102 mg, 0.8 mmol) in CDCl_3_ (0.4 mL) was added dropwise to a stirred solution of (chlorocarbonyl)disulfanyl chloride (**5**) (65 mg, 0.4 mmol) in CDCl_3_ (0.4 mL) at 4 °C, giving the title product **2′** (~65%) [^1^H NMR (300 MHz, CDCl_3_) δ 7.2 (m, 1H), 7.1 (m, 2H), 3.32 (s, 3H), 2.24 (s, 6H), i.e., matching Method A] admixed with (carbamoyl)disulfenamide **6′** (~15%) [^1^H NMR (300 MHz): δ 7.2–7.1 (m, 6H), 3.32 (s, 3H), 3.27 (s, 3H), 2.32 (s, 6H), 2.24 (s, 6H), i.e., matching when this compound was made intentionally], and carbamoyl chloride **9′** (~20%) [^1^H NMR (300 MHz): δ 7.1–7.2 (m, 3H), 3.25 (s, 3H), 2.27 (s, 6H)]. In addition, *N*,2,6-trimethylaniline hydrochloride (1 equiv) was noted at δ 2.79, 2.30, matching [1]. The title product **2′**, in solution at 25 °C, decomposed completely over a period of 7 d to carbamoyl chloride **9′**, with no other species detected spectroscopically at intermediate timepoints. **Method C.** A solution of thiocarbamate **10a [1]** (29.6 mg, 0.1 mmol) in CDCl_3_ (3 mL) was added at 4 °C to a solution of SCl_2_ (20 mg, 0.1 mmol) in CDCl_3_ (3 mL), after which the reaction was maintained at 25 °C for 30 min, by the end of which time the title compound [^1^H NMR: 7.2 (m, 1H), 7.1 (m, 2H), 3.32 (s, 3H), 2.24 (s, 6H)] had formed quantitatively, along with EtCl [^1^H NMR: δ 3.57 (q, *J* = 7.2 Hz, 2H), 1.48 (t, *J* = 7.2 Hz, 3H)] as a co-product. In a separate experiment, the reaction was monitored in real time by ^1^H NMR. Only title product **2′** and co-product EtCl were observed after 5 min; starting **10a** was completely consumed and there was no evidence for any intermediate(s).

**4-Methyl-2(3*H*)-benzo-1,2,4-dithiazinone (3)**. A solution of *N*-methylaniline (643 mg, 6.0 mmol) in CHCl_3_ (12 mL) was added over a period of 15 min to a stirred solution of (chlorocarbonyl)disulfanyl chloride (**5**) (489 mg, 3.0 mmol) in CHCl_3_ (3 mL) at 5 °C. The homogeneous reaction mixture was allowed to warm to 25 °C and stirred for 2 d. The solution was then washed with 1 M aqueous HCl (3 × 10 mL) and brine (10 mL), dried (MgSO_4_), concentrated to ~1 mL and applied to flash column chromatography (4:1 hexanes–EtOAc) to give the pure product as a yellow oil (18 mg, 3%). ^1^H NMR (400 MHz; CDCl_3_): δ 7.51 (dd, *J* = 7.5, 1.5 Hz, 1H), 7.39 (ddd, *J* = 8.3, 7.5, 1.5 Hz, 1H), 7.2–7.1 (m, 2H), 3.53 (s, 3H); ^13^C NMR (101 MHz; CDCl_3_): 170.8, 143.9, 129.1, 125.4, 124.5, 119.5, 35.6. HRMS (ESI) *m*/*z* 219.9864 ([M + Na]^+^, calcd 219.9862), 198.0037 ([M + H]^+^, calcd for C_7_H_14_NO_3_S: 198.0042); 165.0218 ([M − S]^+^, calcd: 165.0248); 136.0218 ([M − CH_3_NS]^+^, calcd 135.9983). This procedure was carried out numerous times throughout the time span of these studies, with some variations in reaction conditions, but overall similar yields and purities. Note that the chromatography typically provided a later-eluting fraction, at a level 2–3 fold the amount of **3**, which was characterized to be pure **4**.

X-ray quality crystals of the title compound were obtained (2015) by dissolving the title product **3** (18 mg) in CH_2_Cl_2_ (10 µL) and adding heptane (20 µL), followed by slow evaporation of the solvent at 5 °C for 10 d; and they were also obtained (2024), by using an 5 mm NMR tube that already contained **3** (15 mg) in CDCl_3_ (0.4 mL), topping it off with CH_2_Cl_2_ (0.1 mL) and heptane (0.4 mL), and transferring to 5 °C where crystals formed after 2 weeks; after 5 weeks, the total volume was 0.25 mL, so more heptane (0.5 mL was added, and a crystal suitable for X-ray analysis was removed one week later.

**3-Methyl-2(3*H*)-benzothiazolone (4)** (CAS 2786-62-1). This compound was first made in 1910 [4], and we have had a sample in our lab since the early 1980s, as described in [6,7]. Because compound **4** arises at several points in the current research, we provide here the relevant spectral information, some of which has not been previously reported. ^1^H NMR (400 MHz, CDCl_3_) δ 7.43 (dd, *J* = 7.8, 1.2 Hz, 1H), 7.33 (td, *J* = 7.8, 1.3 Hz, 1H), 7.17 (td, *J* = 7.6, 1.1 Hz, 1H), 7.04 (dd, *J* = 8.2, 1.1 Hz, 1H), 3.46 (s, 3H); ^13^C NMR (101 MHz, CDCl_3_) δ 170.1, 137.8, 126.4, 123.2, 122.6, 110.4, 29.0.

**(*N*-methyl-*N*-2,6-dimethylphenylamino)(*N*-methyl-*N*-2,6-dimethylphenylcarbamoyl)disulfane** (**6′**). A solution of (chlorocarbonyl)disulfanyl chloride (**5**) (87 mg, 0.53 mmol) in CDCl_3_ (0.29 mL) was added, at 4 °C, to a solution of *N*,2,6-trimethylaniline (0.335 g, 4.64 equiv) in CDCl_3_ (1.2 mL) over 15 min. The solution was then allowed to react overnight at 25 °C. A standard extractive workup (3 × 1 M aqueous HCl) provided, after washing with MeOH, white crystals (74 mg, 77%), mp 165–167 °C. ^1^H NMR (400 MHz, CDCl_3_): δ 7.2–7.1 (m, 3H), 6.97 (s, 3H), 3.32 (s, 3H), 3.27 (s, 3H), 2.32 (s, 6H), 2.24 (s, 6H). ^13^C NMR (101 MHz, CDCl_3_): 167.3, 148.5, 138.8, 137.3, 135.1, 129.1, 128.8, 128.6, 126.4, 47.0, 36.2, 19.2, 17.6. HRMS (ESI) *m*/*z* [M + Na^+^] calcd for C_19_H_24_N_2_OS_2_: 383.1222; found: 383.1222; [M + H^+^] calcd: 361.1403; found: 361.1393; [dimer + Na^+^] calcd: 743.2552; found: 743.2539. Anal. Calcd for C_19_H_24_N_2_OS_2_ (mol wt 360.54): C, 63.30; H, 6.71; N, 7.77. Found: C, 63.00; H, 6.89; N, 7.63.

**Bis(*N*-methyl-*N*-phenylcarbamoyl)trisulfane (11)**. This compound was reported previously in 1986 [3], but the procedures that follow were pursued in the present work (Scheme 2) as ways to further elucidate the chemistry of **2**. **Method A (preferred).** A solution of thiocarbamate **10b** (0.11 g, 0.5 mmol) in CDCl_3_ (5 mL) was added to a freshly prepared stirred solution of (carbamoyl)disulfanyl chloride **2** (~0.5 mmol, ~1 M in CDCl_3_) at 4 °C. After 4.5 h, extractive workup and concentration yielded the title product, which was recrystallized from hexanes, as a white solid (0.11 g, 69%), mp 161–165 °C. ^1^H NMR (500 MHz): δ 7.4 (m, 4H), 7.2 (m, 6H), 3.35 (s, 6H). **Method B.** A solution of (chlorocarbonyl)disulfanyl chloride (**5**) (65 mg, 0.4 mmol) in CDCl_3_ (4 mL) was added to a solution of thiocarbamate **10b** (50 mg, 0.3 mmol) in CDCl_3_ (3 mL) at 4 °C to give (carbamoyl)trithiocarbonyl chloride **15** *in situ*. Next, the standard *N*-methylaniline derivatization procedure, workup, and recrystallization (from hexanes) provided the title product as a white solid (64 mg, 40%), mp 162–164 °C (lit. mp 168–171 °C [3]).

**2-Chlorocyclohexyl (*N*-methyl-*N*-phenylcarbamoyl)disulfane (13). Method A (preferred).** A solution of cyclohexene (40 mg, 0.5 mmol) in CHCl_3_ (0.5 mL) was added slowly to a stirred solution of (chlorocarbonyl)disulfanyl chloride **5** (75 mg, 0.5 mmol) in CHCl_3_ (0.5 mL) at 25 °C. After a further 1 h, the resultant 0.5 M solution, containing carbonyl chloride **14** generated *in situ*, was added to a solution of *N*-methylaniline in CHCl_3_ (1 mL, 2 M, 2 mmol) at 4 °C, and the acylation reaction was conducted for 15 min. The usual extractive workup and concentration provided a yellow oil which was purified by flash chromatography to give a clear oil (35 mg, 22%). ^1^H NMR (300 MHz, CDCl_3_): δ 7.5–7.3 (m, 5H), 4.12 (m, 1H), 3.38 (s, 3H), 3.03 (m, 1H), 2.35 (m, 1H), 2.28–2.21 (m, 1H), 1.74 (m, 2H), 1.64 (m, 2H),1.42 (m, 2H). ^13^C NMR (75 MHz, CDCl_3_): δ 166.5, 141.2, 129.7, 129.0, 128.4, 61.8, 55.7, 39.4, 28.9, 23.2, 22.8. HRMS (ESI): *m*/*z* 338.0422 ([M + Na^+^], calcd for C_14_H_18_ClNOS_2_Na: 338.0416). **Method B.** Freshly prepared **2** in CHCl_3_ (~0.5 mL, 1 M, 0.5 mmol) was added dropwise to a solution of cyclohexene (60 mL, 0.6 mmol) in CHCl_3_ (0.3 mL) at 25 °C. After 1 h reaction, the resultant solution was concentrated at reduced pressure to provide a yellow oil (0.81 g) that included title product **13** (~50%). Pure **13** (29 mg, 15%) was obtained after flash chromatography (3:1 hexanes–EtOH) and gave spectral data matching Method A.

***Tert-*Butyl (*N*,*2*,*6*-trimethyl-*N*-phenylcarbamoyl)trisulfane (16′)**. **Method A (preferred).** A solution of 2-methyl-2-propanethiol (60 μL, 0.5 mmol, slight excess) in CHCl_3_ (3 mL) was added all at once at 4 °C to a 1 M solution of (carbamoyl)disulfanyl chloride **2′** prepared *in situ* from **10a’** [3,6] plus SCl_2_ in CHCl_3_ (0.4 mmol scale; Method C for **2′**). After 1 h at 25 °C, washing with 1 M aqueous HCl (3 × 10 mL), drying (MgSO_4_), concentration, and recrystallization from hot heptane provided the title product as clear crystals (92 mg, 73%), mp 106–108 °C. ^1^H NMR (300 MHz, CDCl_3_): δ 7.23 (t, *J* = 8.1 Hz, 1H) 7.11 (d, *J* = 7.5 Hz, 2H) 3.25 (s, 3H), 2.23 (s, 6H), 1.34 (s, 9H); ^13^C NMR (75 MHz, CDCl_3_): 165.9, 137.8, 137.5, 129.5, 128.9, 49.0, 36.2, 29.7, 17.6. HRMS (ESI): *m*/*z* 338.0691 ([M + Na^+^], calcd for C_14_H_18_ClNOS_2_Na: 338.0683). **Method B.** A solution of 2-methyl-2-propanethiol (81 μL, 7.3 mmol, 1 equiv) in CHCl_3_ (10 mL) was added dropwise over 10 min at 4 °C to a stirred solution of (chlorocarbonyl)disulfanyl chloride (**5**) (1.19 g, 7.3 mmol, 1 equiv) in CHCl_3_ (30 mL). A day later, the resultant 2 M solution of **17** in CHCl_3_ was combined with a 2 M solution of *N*,2,6-trimethylaniline in CHCl_3_ (10 mL), and reacted for 15 min at 25 °C. The standard extractive workup with 1 M aqueous HCl (3 × 10 mL), drying (MgSO_4_), followed by concentration, provided a yellow oil (1.6 g) that comprised principally (~80%) title product **16′**, corresponding to an overall yield of ~70%. Other components of the reaction mixture were not investigated.

**Desulfurization of dithiazinone 3 with triphenylphosphine**. The relevant chemistry is summarized in the text and Scheme 4. Solid triphenylphosphine (29 mg, 0.1 mmol) was added to a stirred solution of substrate **3** (10 mg, 0.05 mmol) in CDCl_3_ (2 mL) at 25 °C. The homogenous reaction mixture was examined within 5 min by NMR, which revealed that dithiazinone **3** (diagnostic singlet at δ 3.52 in ^1^H and 35.6 in ^13^C) had been completely consumed and replaced by thiazolone **4** (diagnostic singlet at δ 3.45 in ^1^H and 29.0 in ^13^C). Meanwhile, ^31^P NMR clearly showed formation of triphenylphosphine sulfide (δ 43.3), along with unreacted triphenylphosphine (δ −5.4), which had been in excess, and relatively little (~7%) triphenylphosphine oxide. In a converse experiment where substrate **3** was in excess over triphenylphosphine, the ^1^H and ^13^C NMR showed the formation of thiazolone **4** along with unreacted **3**, and the ^31^P NMR showed complete conversion of triphenylphosphine to its sulfide, with negligible starting phosphine and/or phosphine oxide by-product (< 2%).

## 4. Summary and Conclusions

The overall premise of this study was to homologate previously described chemistry that had been used successfully to access and characterize (carbamoyl)sulfenyl chlorides (**1**), in a manner that would furnish novel (carbamoyl)disulfanyl chlorides (**2**). This plan included obtaining a number of derivatives of **2** after trapping or quenching with alkenes, thiols, and thiocarbamates. While the parent compounds **2** were quite labile and could not be isolated out of solution (this all in contrast to what was observed with **1**), the aforementioned goals were mostly achieved with the preparation of a series of new products (e.g., **3**, **13**, **15**, **16**, **17**) containing an additional sulfur. Alternative pathways to some of the new products corroborated their structural assignments. Interesting differences were noted between compounds related to unsubstituted *N*-methylaniline, in comparison to the corresponding compounds related to *N*,2,6-trimethylaniline.

Derivative **1** for unsubstituted *N*-methylaniline is known to undergo an intramolecular electrophilic aromatic substitution to provide a heterocyclic product **4** in which a five-membered ring containing a (carbamoyl)sulfenyl moiety is fused onto the arene with which it is coplanar. The present work explored the corresponding reaction of intermediates **2**, and resulted in the discovery of the novel heterocycle **3**. Single crystal X-ray analysis revealed that in **3**, a six-membered ring containing a (carbamoyl)disulfanyl moiety is *not* coplanar with the arene to which it is fused. Instead, the extra sulfur atom in **3** is significantly out of the plane, suggesting that it might be effectively donated to a suitable soft nucleophilic acceptor. We reasoned that extrusion of the extra sulfur in **3** could result in the highly stable **4**, and indeed, this hypothesis was borne out experimentally.

Future work in this field should be directed at developing robust, scalable procedures directed towards **3** and its analogues, as well as further delving into fascinating mechanistic questions raised herein.^13^

## Data Availability

The CIF file for the X-ray diffraction crystal structure of **3** has been deposited at the Cambridge Crystallographic Data Center (CCDC) under accession code 2470096.

## Notes

3 Footnote 14 in [3] drew compound **19**, a putative (carbamoyl)disulfanyl chloride, and spectral data deduced for “**19**” was included in the experimental section for compound **16** (compound numberings from [3]). Thus, it was claimed that freshly generated ROCCl_2_SSS(C=O)N(Me)Ph rapidly reached equilibrium with RO(C=S)Cl + ClSS(C=O)N(Me)Ph. With the benefit of the comprehensive results reported in the present work, we now realize that the species encountered and described briefly in our previous work was *not* (carbamoyl)disulfanyl chloride (**2**), but in fact heterocycle **4** (compound numberings in the current sentence correspond to the text and Schemes of *this* paper).

4 Sulfurization reagents that convert P(III) to P(V)=S, along with the applications of this chemistry for the creation of potential anti-sense drugs based on DNA or RNA, have been reviewed relatively recently [9,10]. Our contributions to this area [11,12] center around ethoxy-1,2,4-dithiazolin-5-one (EDITH) and 1,2,4-dithiazolidine-3,5-dione (DtsNH), both of which (especially EDITH) effect this transformation with extraordinarily high efficiency. For chemical structures of DtsNH and EDITH, please refer to Scheme 5, later in the paper.

5 To put this result in context, the usual *N*-methylaniline assay [6] of (chlorocarbonyl)disulfanyl chloride (**5**), using the secondary amine in excess, rapidly gives (carbamoyl)disulfenamide **6** [3,13] in excellent yield and purity (Scheme 1, top line, right side). For further context, methods described in the text to create **2** and **6** are based on our previous precedents [1,3,6] that use (chlorocarbonyl)sulfenyl chloride (**7**) plus limiting or excess *N*-methylaniline to create (carbamoyl)sulfenamides **1** or **8**, respectively.

6 There can be little doubt that **1** and **2** are intermediates in the reactions of **7** and **5** with excess *N*-methylaniline or *N*,2,6-trimethylaniline to provide **8** and **6**, respectively. The interception of **1** with a *different* secondary amine to create unsymmetrical “crossover” (carbamoyl)sulfenamide products **8**, with one sulfur, has been precedented [1], but the obvious homologation of such reactions in two-sulfur homologues (i.e., **2**, **6**) was not explicitly pursued in the present work.

7 Careful examination of the ^1^H NMR signal that we assign to **2** reveals two closely adjacent singlets, most likely due to the presence of both protonated and non-protonated species. It was not possible to characterize compound **2** by ^13^C NMR, since reaction conditions required for reliable generation of **2** were too dilute to allow acquisition of spectral data on a time frame that preceded further transformations and/or decomposition of **2**. As reported later in this paper, when the *N*-methylaniline moiety of **2** is replaced by an *N*,2,6-trimethylaniline moiety, the resultant **2’** is considerably more stable, and could be characterized by both ^1^H and ^13^C NMR.

8 In contrast, heterocyclization of (carbamoyl)sulfenyl chloride **1** to provide the one-sulfur heterocycle **4**, as reported in [1], was a function of *both* the method of generating **1** and the concentration of this species. In typical experiments, when **1** (1 M **or** 0.1 M) had been generated by reaction of limiting *N*-methylaniline with (chlorocarbonyl)sulfenyl chloride (**7**) in CDCl_3_ at 25 °C, further conversion of **1** to **4** occurred with t_½_~1 h **or** 24 h, respectively.

9 Precedents for chemoselective acidolytic cleavage of a sulfenamide bond are found in [1,2,19], and references cited therein.

10 The reaction of equimolar amounts of thiocarbamate **10b** with SCl_2_, somewhat analogous to the reaction of **10b** with SO_2_Cl_2_ that reliably gave (carbamoyl)sulfenyl chlorides **1** (plus iPrCl and SO_2_) as documented in [1], was reasonably expected to provide (carbamoyl)disulfanyl chloride **2** plus an equiv of iPrCl—especially in view of the fact that reaction of **10b** (2 equiv) with SCl_2_ is a reliable, high-yield route to trisulfane **11** (see [3]).

11 Prior to numerous examples from ref. [2] and from our own laboratory (e.g., [1,3,6]), the reactions of sulfenyl chlorides with *O*-alkyl thiocarbamates to generate carbamoyl disulfide moieties (with concomitant loss of alkyl chloride) were described in 1960 by Harris [20]—hence our reference to “Harris reactions”.

12 Carbamoyl chlorides **9** and **9’** are entirely unreactive with thiols, and react very sluggishly with methanol or *N*-methylaniline (i.e., negligible product formed on “normal” time-scale of quench procedures that are standard in our laboratory as per [1,3,6], and others).

13 When this manuscript was peer reviewed, two separate anonymous referees suggested potentially productive avenues to further develop and extend the new chemistry reported herein. One proposed replacing the chlorine of **2** with fluorine, while another speculated a connection to Bunte salts. We believe that compelling cases can be made to explore these and other avenues, and hope that other laboratories will rise to these challenges.

## Supplementary Materials

The following supporting information can be downloaded at: https://www.mdpi.com/article/10.3390/molecules30193892/s1, Tables S1–S7 provide X-ray data for **3**. Figures S1 and S2 show packing and nonconvalent interactions in the crystal structure. Figure S3 shows ^1^H NMR monitoring of the reaction of *N*-methylaniline (NMA) with (chlorocarbonyl)disulfanyl chloride (**5**) in a 2:1 ratio, and in the presence of hexamethylbenzene (HMB) as an internal reference. Figures S4–S16 are ^1^H and ^13^C NMR spectra for compounds **2′**, **3**, **4**, **6′**, **13** and **16′**. Figures S17–S22 are ^1^H, ^13^C, and ^31^P NMR spectra for the reactions of compound **3** with excess and limiting triphenylphosphine.

## Abbreviations

The following abbreviations are used in this manuscript:

DtsNH	1,2,4-dithiazolidine-3,5-dione
EDITH	3-ethoxy-1,2,4-dithiazolin-5-one
HMB	hexamethylbenzene
NMA	*N*-methylaniline
PyDITCN	1,3-dimethyl-5*H*-pyrazolo [3,4-*e*][1,2,4]dithiazine-3-carbonitrile
