# Supplementary material for: Synthetic Routes to, Stabilities and Transformations of, and Characterization of (Carbamoyl)disulfanyl Chlorides and Related Compounds,
Source: Molecules. 2025 Sep 26;30(19):3892. doi: 10.3390/molecules30193892 (PMC12525739; doi:10.3390/molecules30193892)

Supplementary Materials

Synthetic Routes to, Stabilities and Transformations of, and Characterization of (Carbamoyl)disulfanyl Chlorides and Related Compounds

Phillip T. Goldblatt [1], Tracy R. Thompson [1,2], William W. Brennessel [3], Thomas G. Smith [1], Alex M. Schrader [1], Erik S. Goebel [1,†], Madeleine J. Henley [1], Alex Lovstedt [1], Victor G. Young, Jr. [1] and George Barany [1,*]

Contents

Table S1. Crystal data and structure refinement for 4-methyl-2(3*H*)-benzo-1,2,4-dithiazinone (**3**) S3

Table S2. Atomic coordinates (x 10^4) and equivalent isotropic displacement parameters (Å^2 x 10^3) for 4-methyl-2(3*H*)-benzo-1,2,4-dithiazinone (**3**) S4

Table S3. Anisotropic displacement parameters (Å^2 x 10^3) for 4-methyl-2(3*H*)-benzo-1,2,4-dithiazinone (**3**) S4

Table S4. Bond lengths [Å] and angles [°] for 4-methyl-2(3*H*)-benzo-1,2,4-dithiazinone (**3**) S5

Table S5. Hydrogen coordinates (x 10^4) and isotropic displacement parameters (Å^2 x 10^3) for 4-methyl-2(3*H*)-benzo-1,2,4-dithiazinone (**3**) S6

Table S6. Torsion angles [°] for 4-methyl-2(3*H*)-benzo-1,2,4-dithiazinone (**3**) S6

Table S7. Hydrogen bonds and close contacts for 4-methyl-2(3*H*)-benzo-1,2,4-dithiazinone (**3**) [Å and °] S6

Figure S1. Unit cell packing of 4-methyl-2(3*H*)-benzo-1,2,4-dithiazinone (**3**) viewed along the *a* axis S7

Figure S2. Exact packing of the molecules of 4-methyl-2(3*H*)-benzo-1,2,4-dithiazinone (**3**), which may be due to weak C–H…O linkages along the [001] direction S7

Figure S3. ^{1}H NMR monitoring of the reaction of *N*-methylaniline (NMA) with (chlorocarbonyl)disulfanyl chloride (**5**) in a 2:1 ratio, and in the presence of hexamethylbenzene (HMB) as an internal reference, in $CDCl_3$ at 25°C, after ~10 min (400 MHz) S8

Figure S4. ^{1}H NMR of (*N*,2,6-trimethylphenylcarbamoyl)disulfanyl chloride (**2′**) for Method A, admixed with (*N*,2,6-trimethylphenylcarbamoyl) chloride (**9′**) in $CDCl_3$ (300 MHz) S9

Figure S5. ^{13}C NMR of (*N*,2,6-trimethylphenylcarbamoyl)disulfanyl chloride (**2′**) for Method A, admixed with (*N*,2,6-trimethylphenylcarbamoyl) chloride (**9′**) in $CDCl_3$ (75 MHz) S10

Figure S6. ^{1}H NMR of (*N*,2,6-trimethylphenylcarbamoyl)disulfanyl chloride (**2′**) for Method C, admixed with ethyl chloride in $CDCl_3$ (300 MHz) S11

Figure S7. ^{1}H NMR of 4-methyl-2(3*H*)-benzo-1,2,4-dithiazinone (**3**) (400 MHz) S12

Figure S8. ^{13}C NMR of 4-methyl-2(3*H*)-benzo-1,2,4-dithiazinone (**3**) (101 MHz) S13

Figure S9. ^{1}H NMR of authentic 3-methyl-2(3*H*)-benzothiazolone (**4**) (400 MHz) S14

Figure S10. ^{13}C NMR of authentic 3-methyl-2(3*H*)-benzothiazolone (**4**) (400 MHz) S15

Figure S11. ^{1}H NMR of (*N*-methyl-*N*-2,6-dimethylphenylamino)(*N*-methyl-*N*-2,6-dimethylphenylcarbamoyl)disulfane (**6′**) (500 MHz) S16

Figure S12. ^{13}C NMR of (*N*-methyl-*N*-2,6-dimethylphenylamino)(*N*-methyl-*N*-2,6-dimethylphenylcarbamoyl)disulfane (**6′**) (126 MHz) S17

Figure S13. ^{1}H NMR of 2-chlorocyclohexyl (*N*-methyl-*N*-phenylcarbamoyl)disulfane (**13**) (300 MHz) S18

Figure S14. ^{13}C NMR of 2-chlorocyclohexyl (*N*-methyl-*N*-phenylcarbamoyl)disulfane (**13**) (75 MHz) S19

Figure S15. ^{1}H NMR of *tert*-butyl (*N,2,6*-trimethyl-*N*-phenylcarbamoyl)trisulfane (**16′**) (300 MHz) S20

Figure S16. ^{13}C NMR of *tert*-butyl (*N,2,6*-trimethyl-*N*-phenylcarbamoyl)trisulfane (**16′**) (75 MHz) S21

Figure S17. ^{1}H NMR of 4-methyl-2(*3H*)-benzo-1,2,4-dithiazinone (**3**) after treatment with excess triphenylphosphine, showing quantitative conversion to 3-methyl-2(3*H*)-benzothiazolone (**4**) (400 MHz) S22

Figure S18. ^{13}C NMR of 4-methyl-2(*3H*)-benzo-1,2,4-dithiazinone (**3**) after treatment with excess triphenylphosphine, showing quantitative conversion to 3-methyl-2(3*H*)-benzothiazolone (**4**) (101 MHz) S23

Figure S19. ^{31}P NMR of 4-methyl-2(*3H*)-benzo-1,2,4-dithiazinone (**3**) after treatment with excess triphenylphosphine, showing quantitative conversion to 3-methyl-2(3*H*)-benzothiazolone (**4**) (162 MHz) S24

Figure S20. ^{1}H NMR of 4-methyl-2(*3H*)-benzo-1,2,4-dithiazinone (**3**) after treatment with limiting triphenylphosphine, showing quantitative conversion to triphenylphosphine sulfide (400 MHz) S25

Figure S21. ^{13}C NMR of 4-methyl-2(*3H*)-benzo-1,2,4-dithiazinone (**3**) after treatment with limiting triphenylphosphine, showing quantitative conversion to triphenylphosphine sulfide (100 MHz) S26

Figure S22. ^{31}P NMR of 4-methyl-2(*3H*)-benzo-1,2,4-dithiazinone (**3**) after treatment with limiting triphenylphosphine, showing quantitative conversion to triphenylphosphine sulfide (162 MHz) S27

Table S1. Crystal data and structure refinement for 4-methyl-2(*3H*)-benzo-1,2,4-dithiazinone (**3**)

Empirical formula	$C_8H_7NOS_2$	
Formula weight (*M*)	197.27 g/mol	
Temperature (*T*)	100(2) K	
Wavelength	0.71073 Å	
Crystal system	monoclinic	
Space group	*Cc* (no. 9)	
Unit cell dimensions	a = 7.6278(4) Å	α = 90°
	b = 15.6732(8) Å	β = 115.983(2)°
	c = 7.8137(4) Å	γ = 90°
Volume (*V*)	839.72(8) $Å^3$	
Z	4	
Density (calculated)	1.560 g/cm^3	
Absorption coefficient μ(MoKα)	0.578 mm^{-1}	
F(000)	408	
Crystal color, morphology	yellow, block	
Crystal size	0.250 x 0.170 x 0.100 mm^3	
Θ range for data collection	2.599 to 28.301°	
Index ranges	$-10 \le h \le 10$, $-20 \le k \le 20$, $-10 \le l \le 10$	
Reflections collected	10905	
Independent reflections	2076 [R_{int} = 0.0256]	
Observed reflections	2053	
Completeness to θ = 25.242°	100.0%	
Absorption correction	Multi-scan	
Max. and min. transmission	0.7457 and 0.6994	
Refinement method	Full-matrix least-squares on F^2	
Data / restraints / parameters	2076 / 2 / 110	
Goodness-of-fit on F^2	1.072	
Final *R* indices [$I > 2\sigma(I)$]	$R1$ = 0.0181, $wR2$ = 0.0481	
R indices (all data)	$R1$ = 0.0183, $wR2$ = 0.0484	
Absolute structure parameter	– 0.03(2)	
Largest diff. peak and hole	0.305 and – 0.141 e.$Å^{-3}$	

Table S2. Atomic coordinates (x 10^4) and equivalent isotropic displacement parameters (Å^2 x 10^3) for 4-methyl-2(*3H*)-benzo-1,2,4-dithiazinone (**3**)

	x	y	z	U_{eq}
S1	5893(1)	6254(1)	3624(1)	14(1)
S2	7566(1)	7170(1)	5527(1)	13(1)
O1	3815(2)	5235(1)	4652(2)	20(1)
N1	3777(2)	6611(1)	5565(2)	12(1)
C1	4349(3)	5972(1)	4736(2)	13(1)
C2	2447(3)	6337(1)	6386(3)	16(1)
C11	3970(2)	7504(1)	5337(2)	11(1)
C12	2452(3)	8052(1)	5156(3)	14(1)
C13	2625(3)	8928(1)	4999(3)	17(1)
C14	4297(3)	9271(1)	4988(3)	18(1)
C15	5805(3)	8737(1)	5127(3)	14(1)
C16	5647(3)	7856(1)	5307(2)	12(1)

U_{eq} is defined as one third of the trace of the orthogonalized U_{ij} tensor.

Table S3. Anisotropic displacement parameters (Å^2 x 10^3) for 4-methyl-2(*3H*)-benzo-1,2,4-dithiazinone (**3**)

	U_{11}	U_{22}	U_{33}	U_{23}	U_{13}	U_{12}
S1	16(1)	13(1)	16(1)	–2(1)	10(1)	0(1)
S2	10(1)	15(1)	15(1)	0(1)	6(1)	1(1)
O1	24(1)	12(1)	28(1)	–2(1)	16(1)	–2(1)
N1	15(1)	10(1)	15(1)	0(1)	9(1)	–1(1)
C1	13(1)	14(1)	14(1)	0(1)	6(1)	1(1)
C2	19(1)	13(1)	23(1)	2(1)	15(1)	–1(1)
C11	13(1)	10(1)	12(1)	1(1)	6(1)	1(1)
C12	12(1)	14(1)	18(1)	0(1)	7(1)	1(1)
C13	17(1)	14(1)	21(1)	0(1)	10(1)	4(1)
C14	22(1)	10(1)	21(1)	–1(1)	9(1)	–1(1)
C15	14(1)	13(1)	16(1)	–2(1)	6(1)	–3(1)
C16	11(1)	12(1)	12(1)	–1(1)	4(1)	2(1)

The anisotropic displacement factor exponent takes the form: $-2p^2[\, h^2 a^{*2}U_{11} + ... + 2\, h\, k\, a^*\, b^*\, U_{12}\,]$.

Table S4. Bond lengths [Å] and angles [°] for 4–methyl–2(*3H*)–benzo–1,2,4–dithiazinone (**3**)

Bond lengths [Å]	
S1–C1	1.7976(18)
S1–S2	2.0580(6)
S2–C16	1.7627(18)
O1–C1	1.217(2)
N1–C1	1.363(2)
N1–C11	1.427(2)
N1–C2	1.482(2)
C2–H2A	0.9800
C2–H2B	0.9800
C2–H2C	0.9800
C11–C12	1.398(2)
C11–C16	1.403(2)
C12–C13	1.390(2)
C12–H12A	0.9500
C13–C14	1.388(3)
C13–H13A	0.9500
C14–C15	1.388(3)
C14–H14A	0.9500
C15–C16	1.398(2)
C15–H15A	0.9500

Angles [°]	
C1–S1–S2	98.78(6)
C16–S2–S1	97.43(6)
C1–N1–C11	126.11(15)
C1–N1–C2	114.57(15)
C11–N1–C2	117.70(14)
O1–C1–N1	123.55(17)
O1–C1–S1	118.84(14)
N1–C1–S1	117.55(13)
N1–C2–H2A	109.5
N1–C2–H2B	109.5
H2A–C2–H2B	109.5
N1–C2–H2C	109.5
H2A–C2–H2C	109.5
H2B–C2–H2C	109.5
C12–C11–C16	118.55(16)
C12–C11–N1	119.31(15)
C16–C11–N1	122.13(15)
C13–C12–C11	120.60(17)
C13–C12–H12A	119.7
C11–C12–H12A	119.7
C14–C13–C12	120.48(17)
C14–C13–H13A	119.8
C12–C13–H13A	119.8
C13–C14–C15	119.83(17)
C13–C14–H14A	120.1
C15–C14–H14A	120.1
C14–C15–C16	119.91(18)
C14–C15–H15A	120.0
C16–C15–H15A	120.0
C15–C16–C11	120.61(17)
C15–C16–S2	120.50(15)
C11–C16–S2	118.89(13)

Table S5. Hydrogen coordinates (x 10^4) and isotropic displacement parameters (Å^2 x 10^3) for 4-methyl-2(*3H*)-benzo-1,2,4-dithiazinone (**3**)

	x	y	z	U(eq)
H2A	2830	5769	6952	24
H2B	1107	6317	5380	24
H2C	2525	6744	7369	24
H12A	1291	7823	5140	17
H13A	1591	9294	4897	20
H14A	4409	9871	4887	21
H15A	6943	8969	5101	17

Table S6. Torsion angles [°] for 4–methyl–2(*3H*)–benzo–1,2,4–dithiazinone (**3**)

C1–S1–S2–C16	–58.36(8)	C11–C12–C13–C14	1.0(3)
C11–N1–C1–O1	–164.59(17)	C12–C13–C14–C15	0.3(3)
C2–N1–C1–O1	0.5(3)	C13–C14–C15–C16	–1.0(3)
C11–N1–C1–S1	12.7(2)	C14–C15–C16–C11	0.4(3)
C2–N1–C1–S1	177.75(13)	C14–C15–C16–S2	–178.88(14)
S2–S1–C1–O1	–145.56(15)	C12–C11–C16–C15	1.0(2)
S2–S1–C1–N1	37.05(14)	N1–C11–C16–C15	–178.45(15)
C1–N1–C11–C12	139.71(18)	C12–C11–C16–S2	–179.79(12)
C2–N1–C11–C12	–25.0(2)	N1–C11–C16–S2	0.8(2)
C1–N1–C11–C16	–40.9(2)	S1–S2–C16–C15	–134.98(14)
C2–N1–C11–C16	154.45(17)	S1–S2–C16–C11	45.77(14)
C16–C11–C12–C13	–1.7(3)		
N1–C11–C12–C13	177.77(15)		

Table S7. Hydrogen bonds and close contacts for 4-methyl-2(*3H*)-benzo-1,2,4-dithiazinone (**3**) [Å and °]

D–H...A	d(D–H)	d(H...A)	d(D...A)	<(DHA)
C2–H2A...O1#1	0.98	2.47	3.367(2)	152.0

Symmetry transformations used to generate equivalent atoms: #1 x, –y+1,z+1/2

Figure S1. Unit cell packing of 4-methyl-2(3*H*)-benzo-1,2,4-dithiazinone (**3**) viewed along the *a* axis

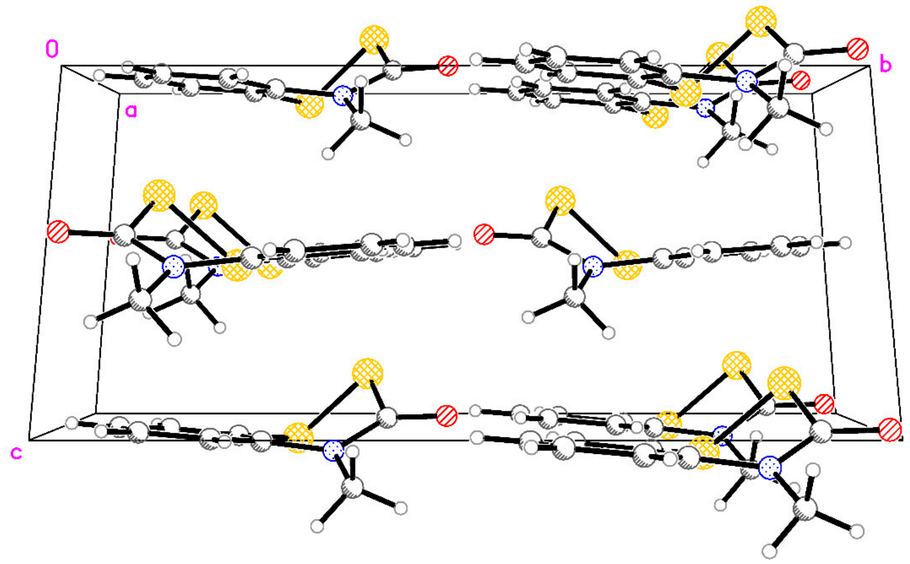

Figure S2. Exact packing of the molecules of 4-methyl-2(3*H*)-benzo-1,2,4-dithiazinone (**3**), which may be due to weak C–H…O linkages along the [001] direction

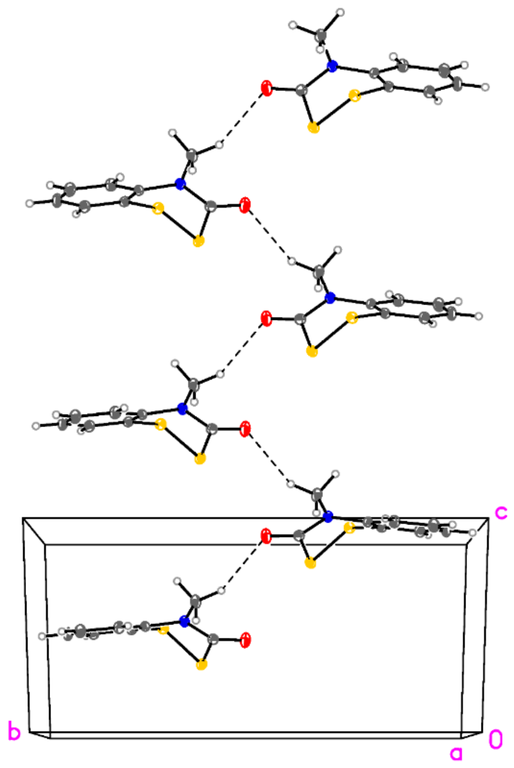

Weak interactions between methyl C–H donors and O=C acceptors are suggested by C…O distances of 3.367(2) Å and C–H…O angles of 152°

Figure S3. ^{1}H NMR monitoring of the reaction of *N*-methylaniline (NMA) with (chlorocarbonyl)disulfanyl chloride (**5**) in a 2:1 ratio, and in the presence of hexamethylbenzene (HMB) as an internal reference, in $CDCl_3$ at 25°C, after ~10 min (400 MHz)

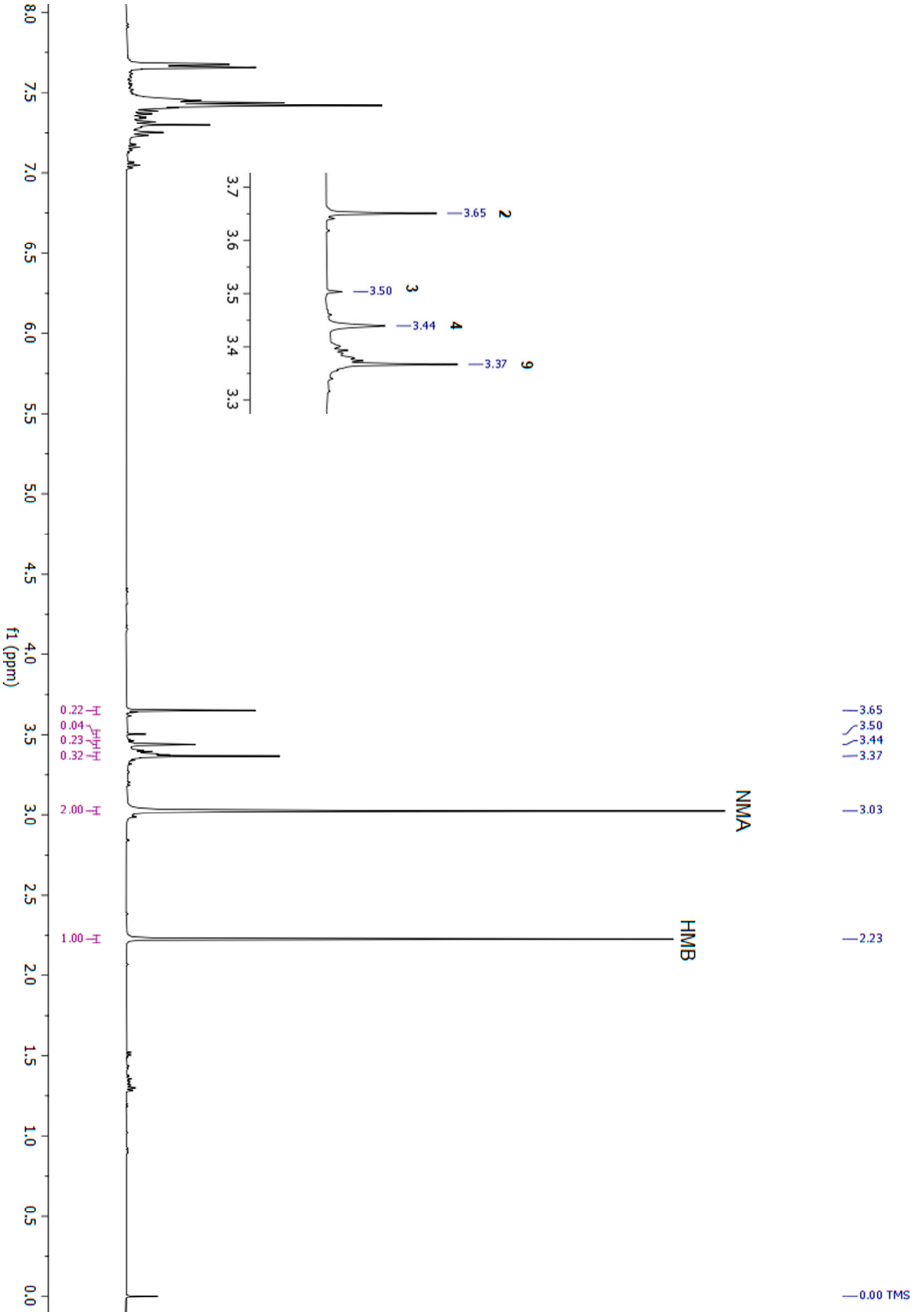

Figure S4. ^{1}H NMR of (*N*,2,6-trimethylphenylcarbamoyl)disulfanyl chloride (**2′**) for Method A, admixed with (*N*,2,6-trimethylphenylcarbamoyl) chloride (**9′**) in $CDCl_3$ (300 MHz)

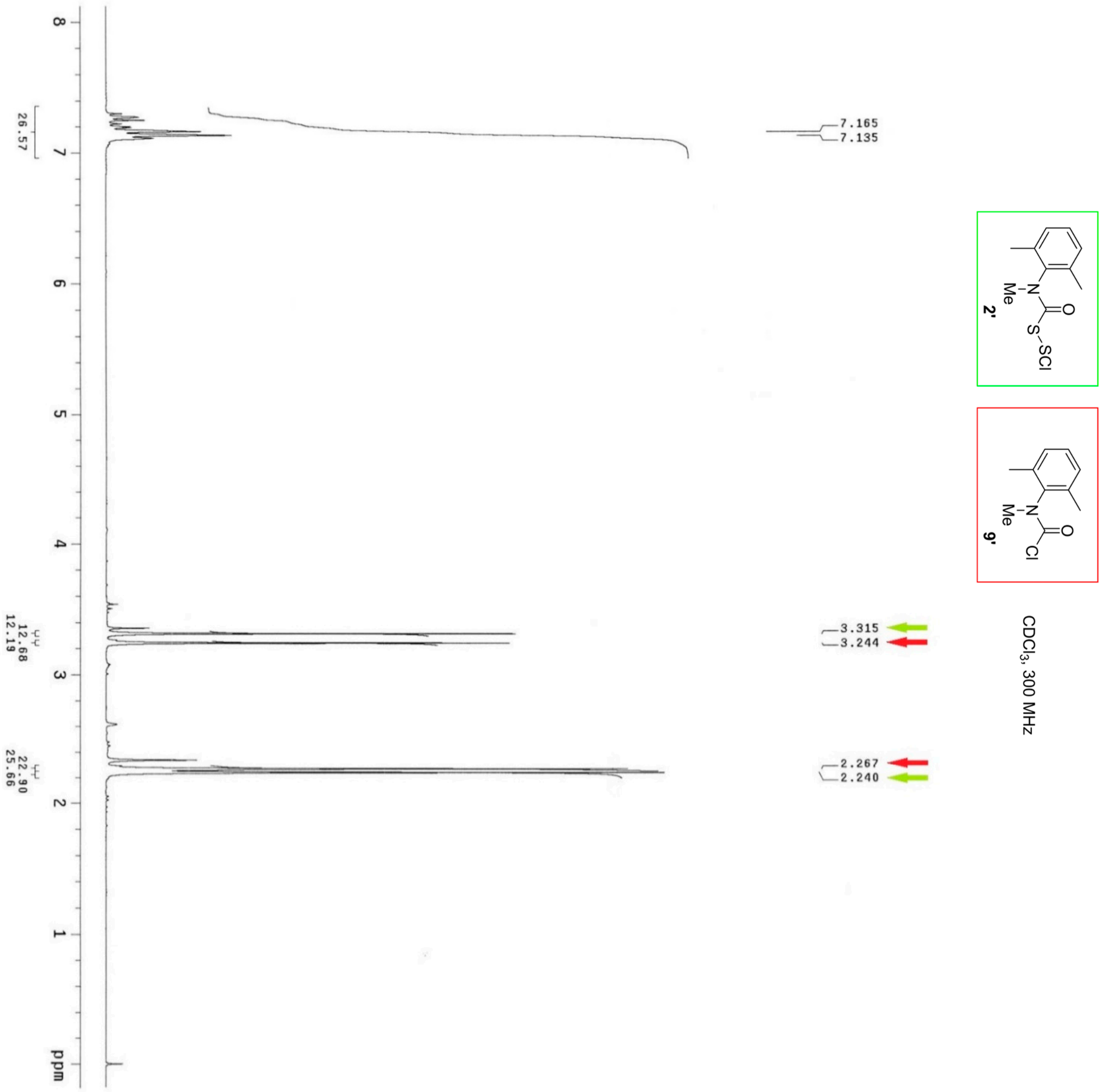

Figure S5. ^{13}C NMR of (*N*,2,6-trimethylphenylcarbamoyl)disulfanyl chloride (**2′**) for Method A, admixed with (*N*,2,6-trimethylphenylcarbamoyl) chloride (**9′**) in $CDCl_3$ (75 MHz)

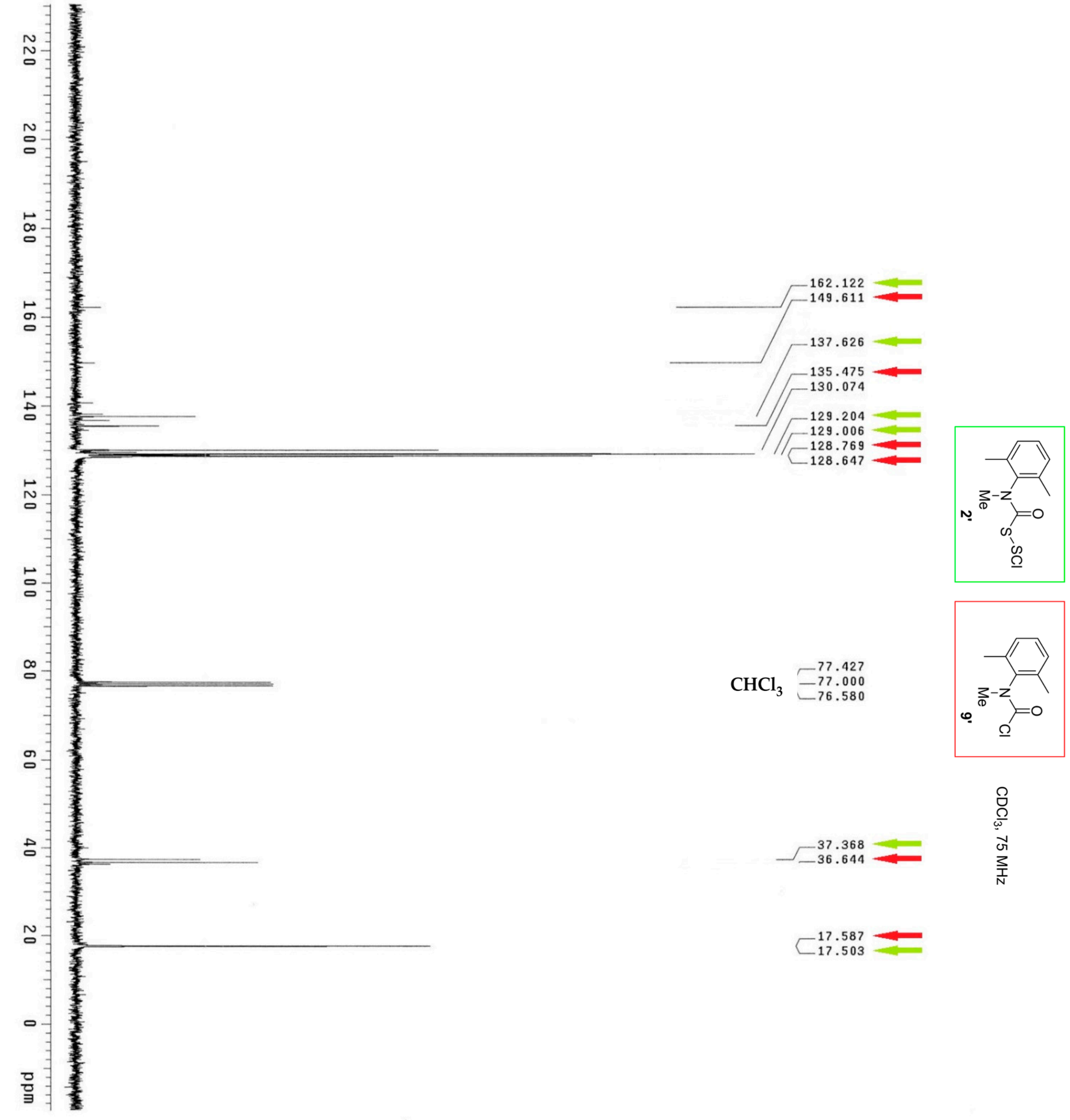

Figure S6. ^{1}H NMR of (*N*,2,6-trimethylphenylcarbamoyl)disulfanyl chloride (**2'**) for Method C, admixed with ethyl chloride in $CDCl_3$ (300 MHz)

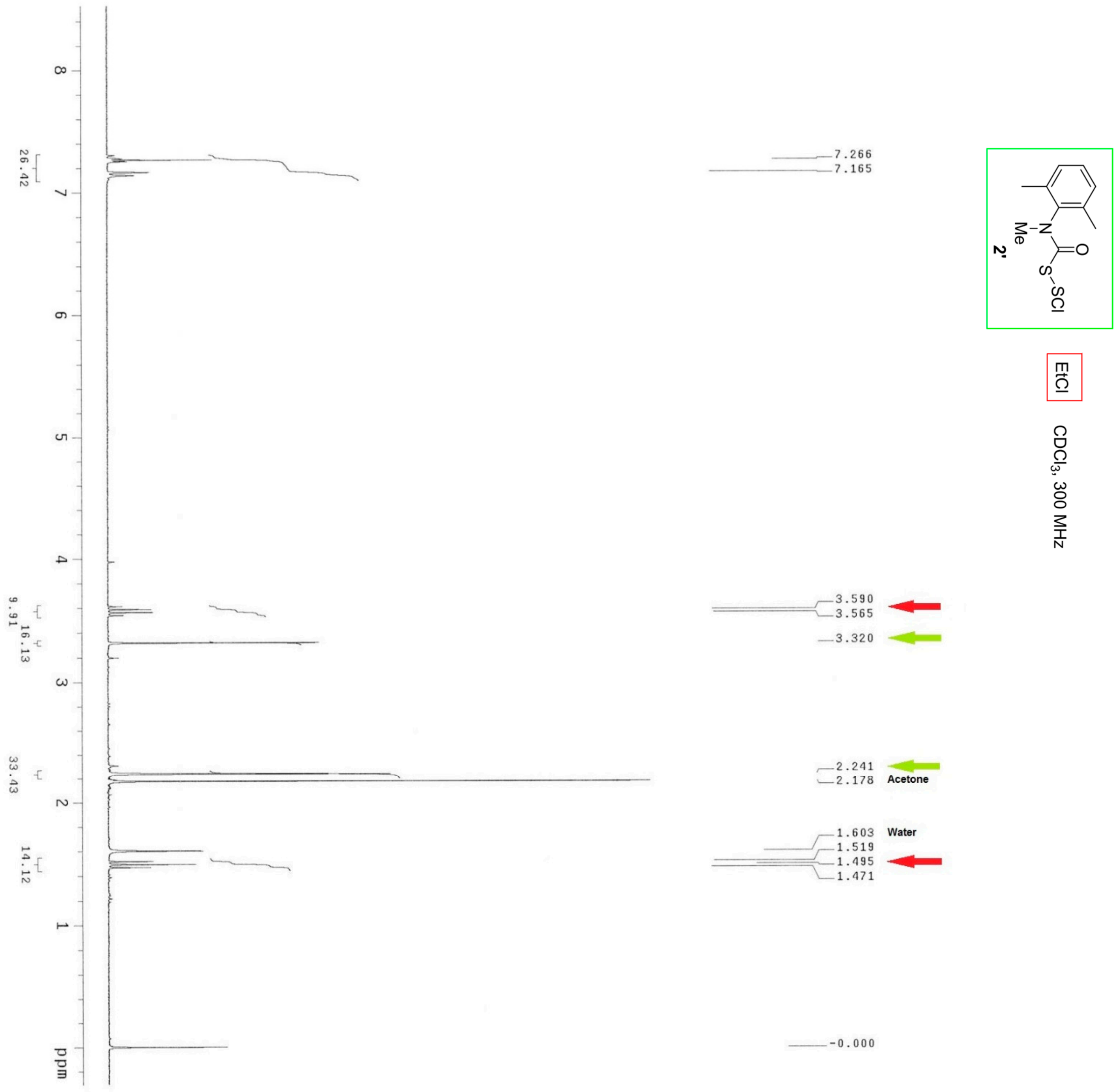

Figure S7. ^{1}H NMR of 4-methyl-2(*3H*)-benzo-1,2,4-dithiazinone (**3**) (400 MHz)

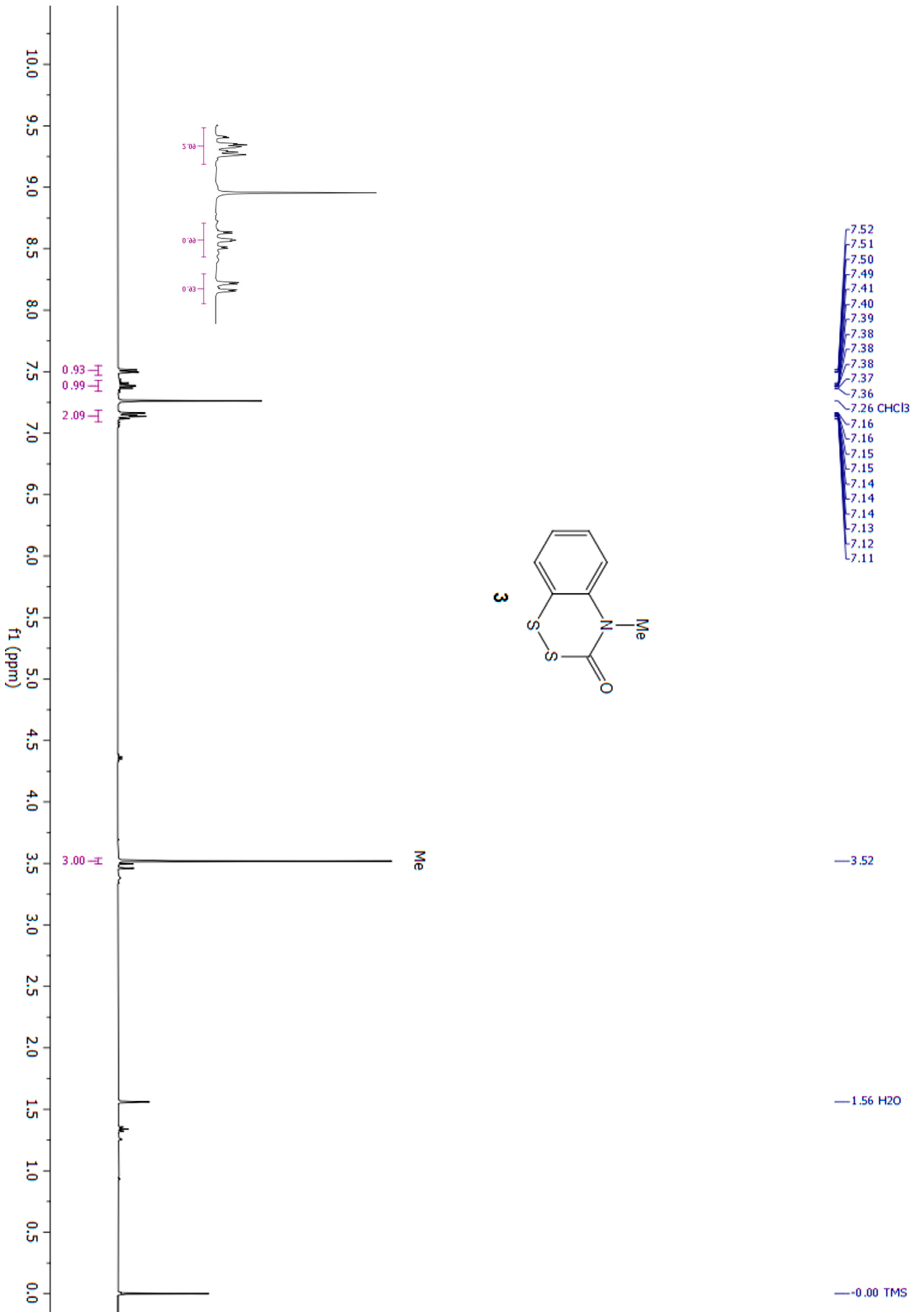

Figure S8. ¹³C NMR of 4-methyl-2(*3H*)-benzo-1,2,4-dithiazinone (**3**) (101 MHz).

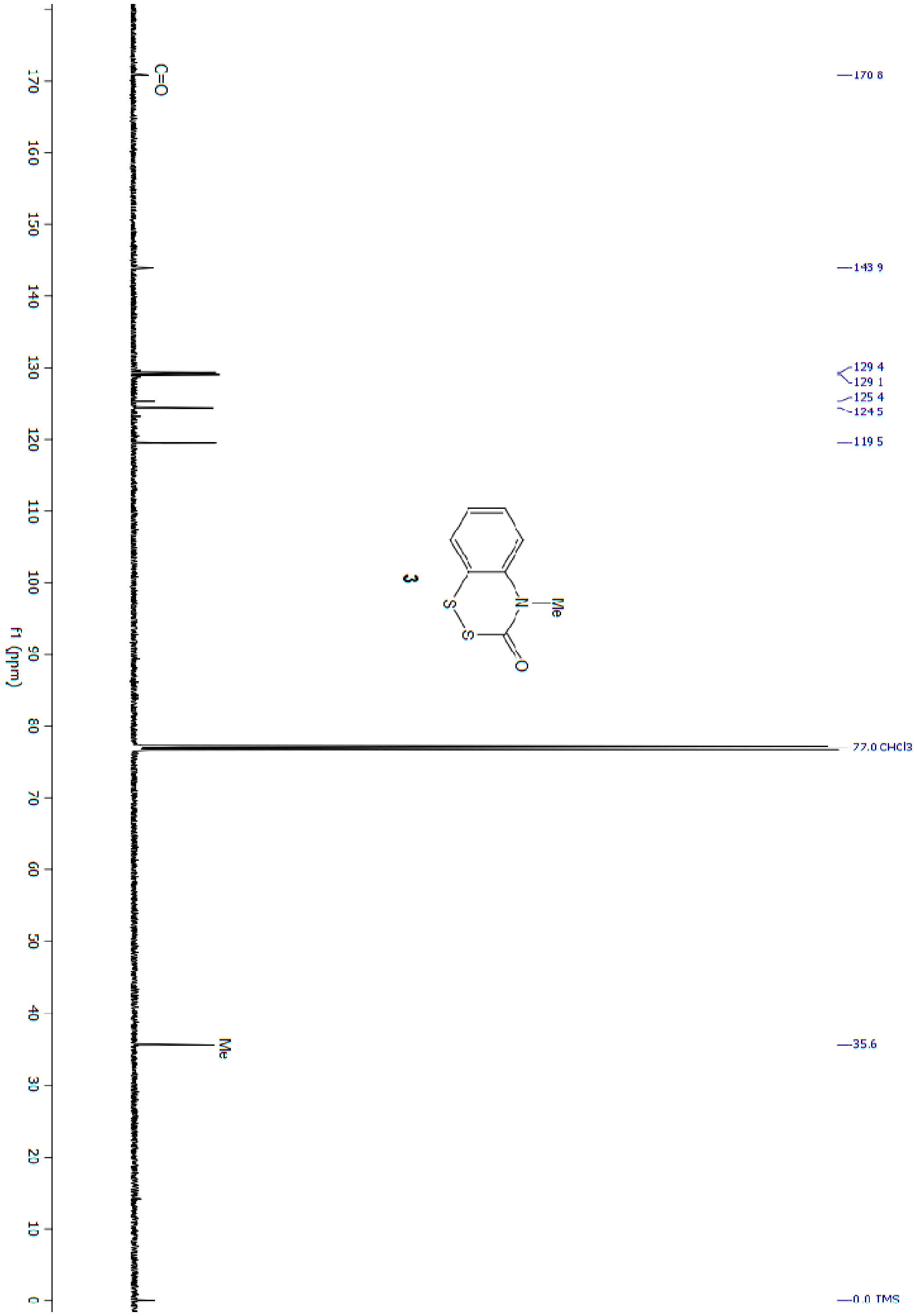

Figure S9. ^{1}H NMR of authentic 3-methyl-2(3*H*)-benzothiazolone (**4**) (400 MHz)

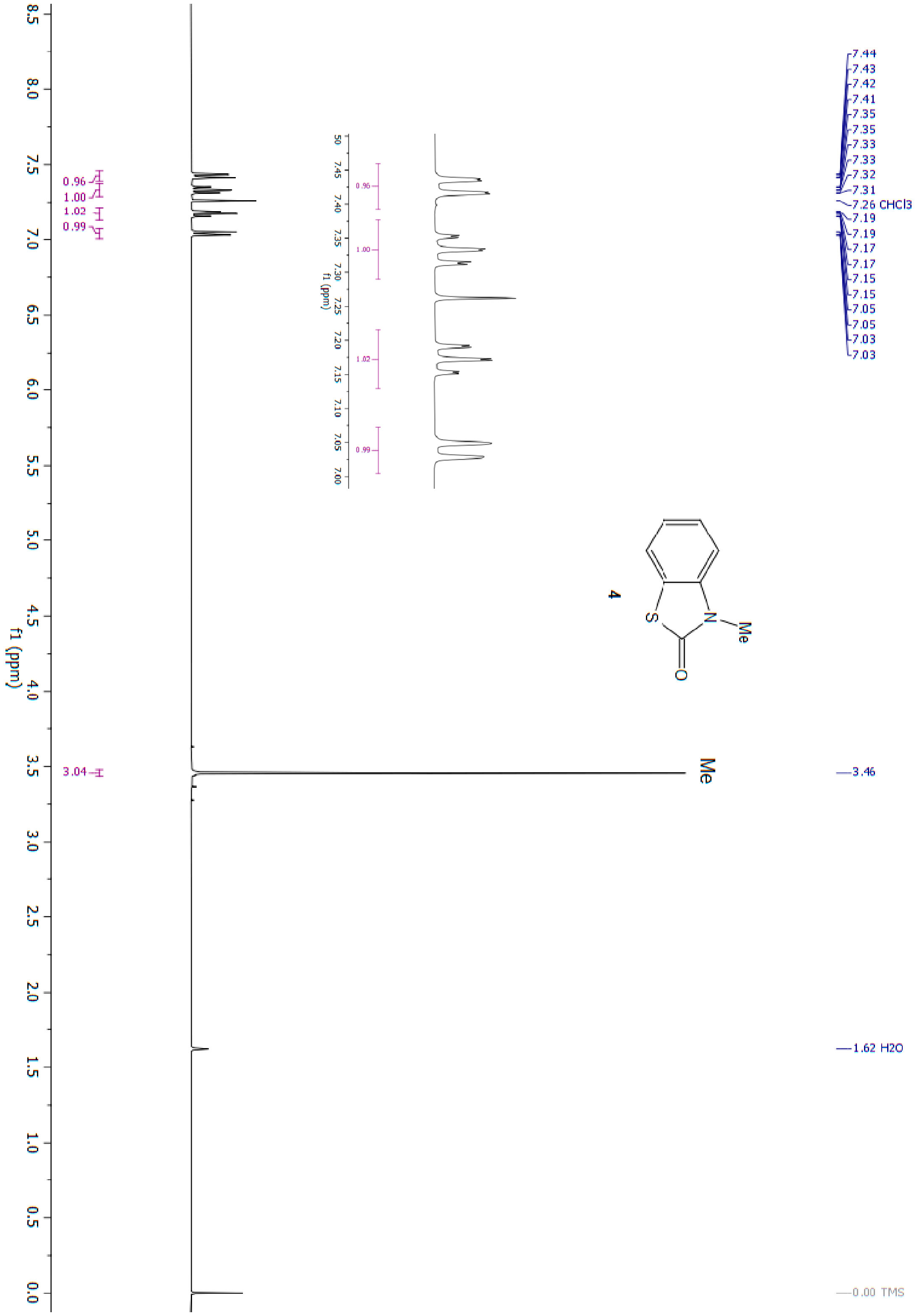

Figure S10. ^{13}C NMR of authentic 3-methyl-2(3*H*)-benzothiazolone (**4**) (101 MHz)

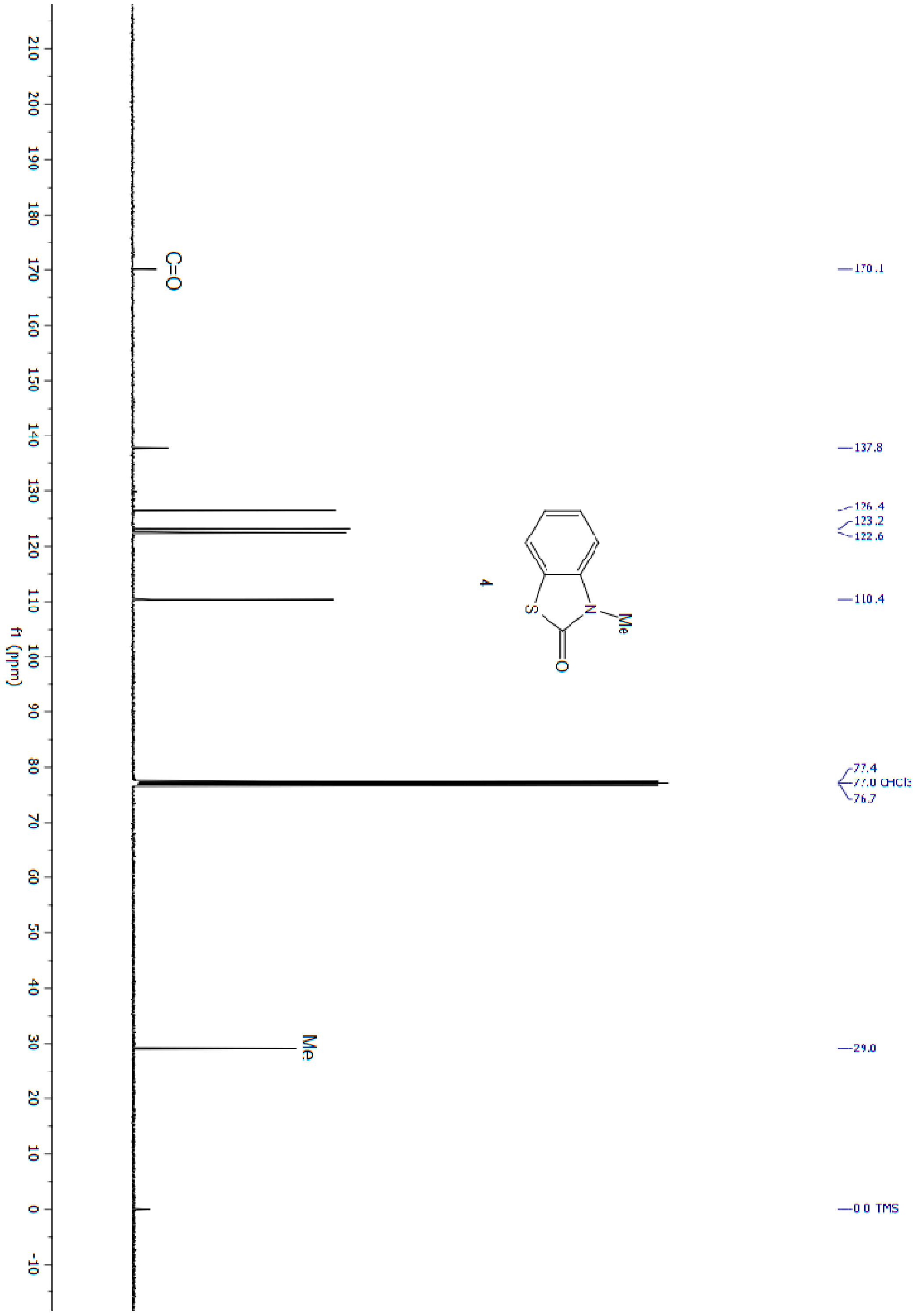

Figure S11. ¹H NMR of (*N*-methyl-*N*-2,6-dimethylphenylamino)(*N*-methyl-*N*-2,6-dimethylphenylcarbamoyl)disulfane (**6’**) (500 MHz)

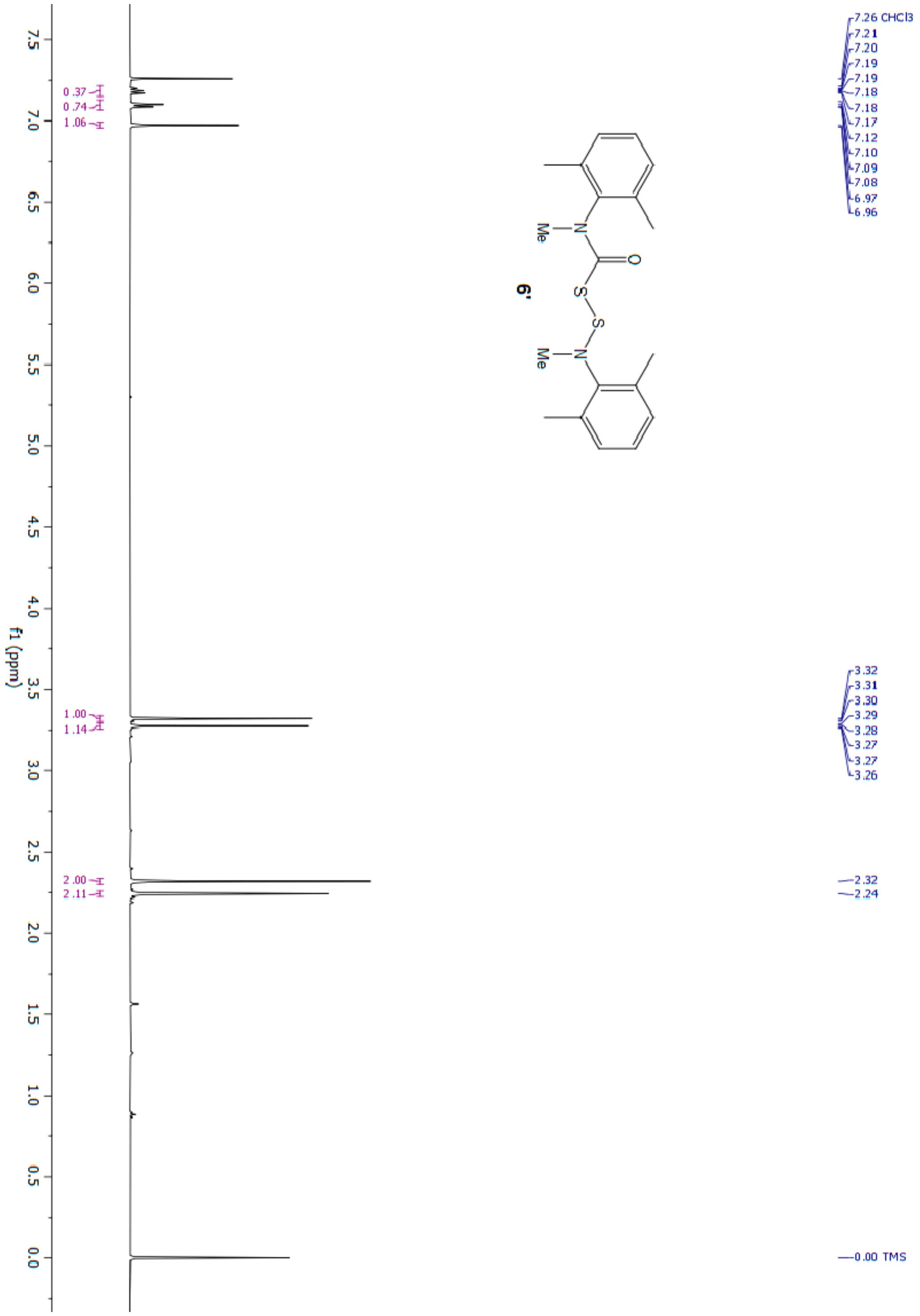

Figure S12. ^{13}C NMR of (*N*-methyl-*N*-2,6-dimethylphenylamino)(*N*-methyl-*N*-2,6-dimethylphenylcarbamoyl)disulfane (**6'**) (126 MHz)

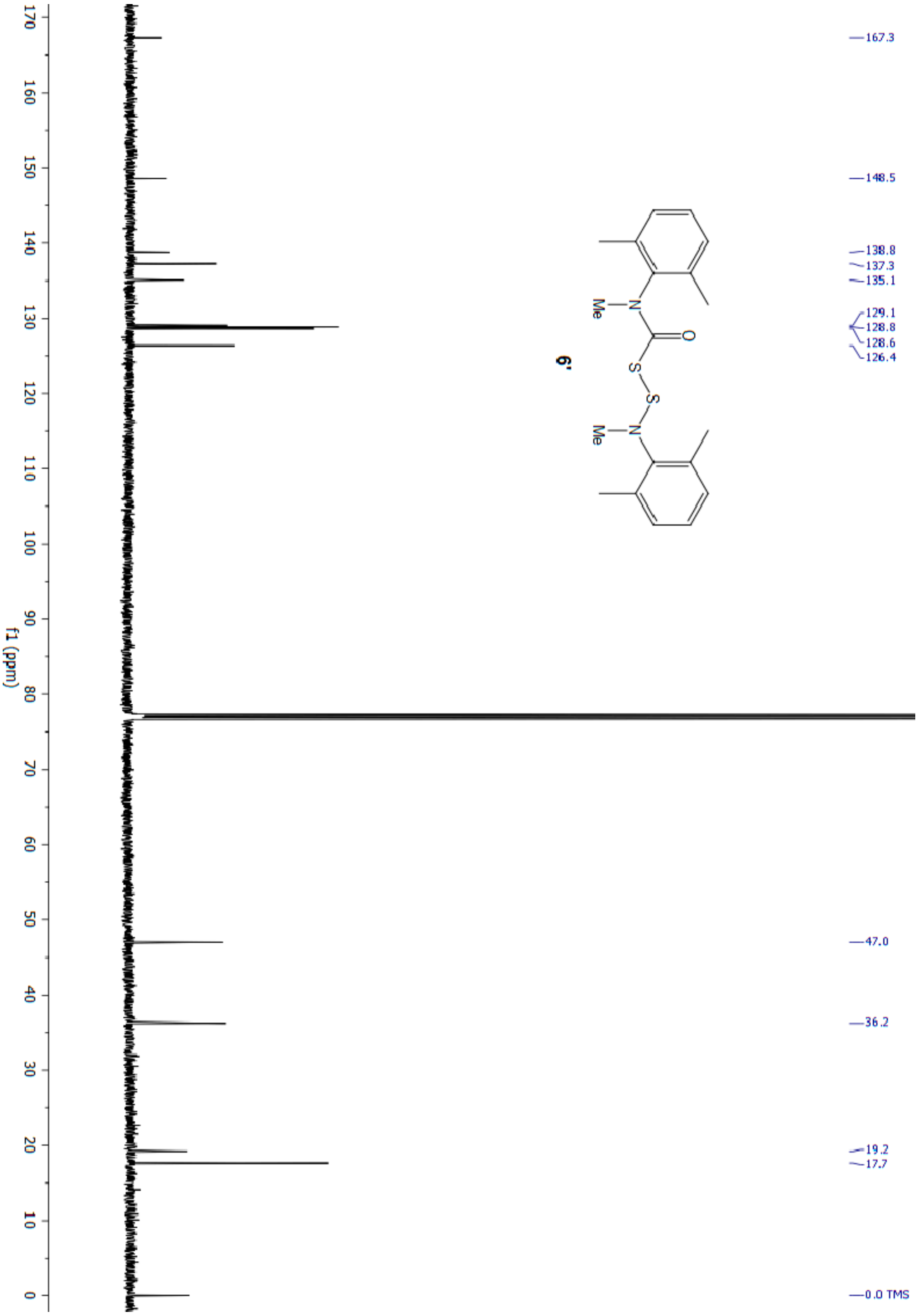

Figure S13. ^{1}H NMR of 2-chlorocyclohexyl (*N*-methyl-*N*-phenylcarbamoyl)disulfane (**13**) (300 MHz)

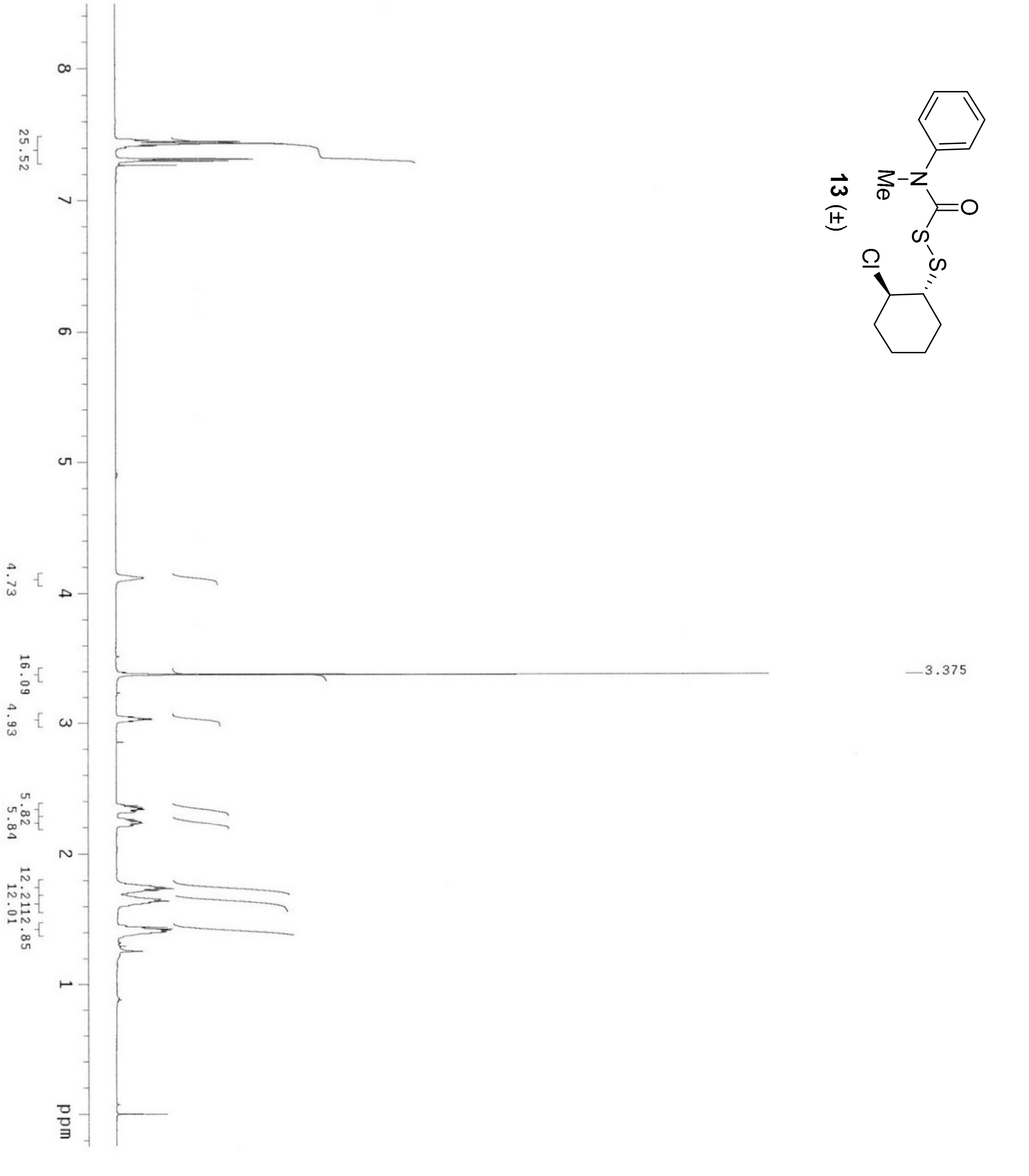

Figure S14. ^{13}C NMR of 2-chlorocyclohexyl (*N*-methyl-*N*-phenylcarbamoyl)disulfane (**13**) (75 MHz)

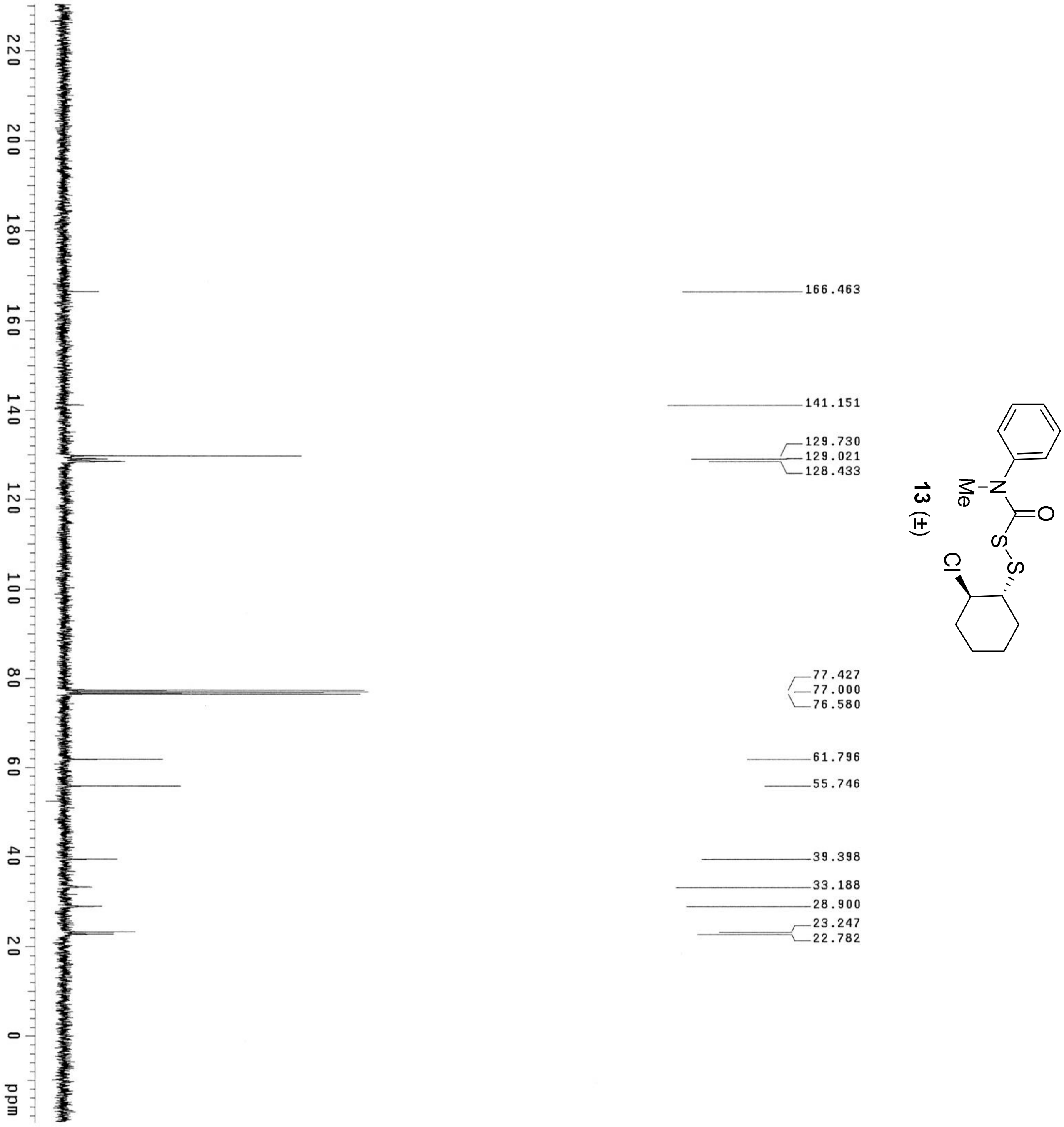

Figure S15. ¹H NMR of *tert*-butyl (*N,2,6*-trimethyl-*N*-phenylcarbamoyl)trisulfane (**16'**) (300 MHz)

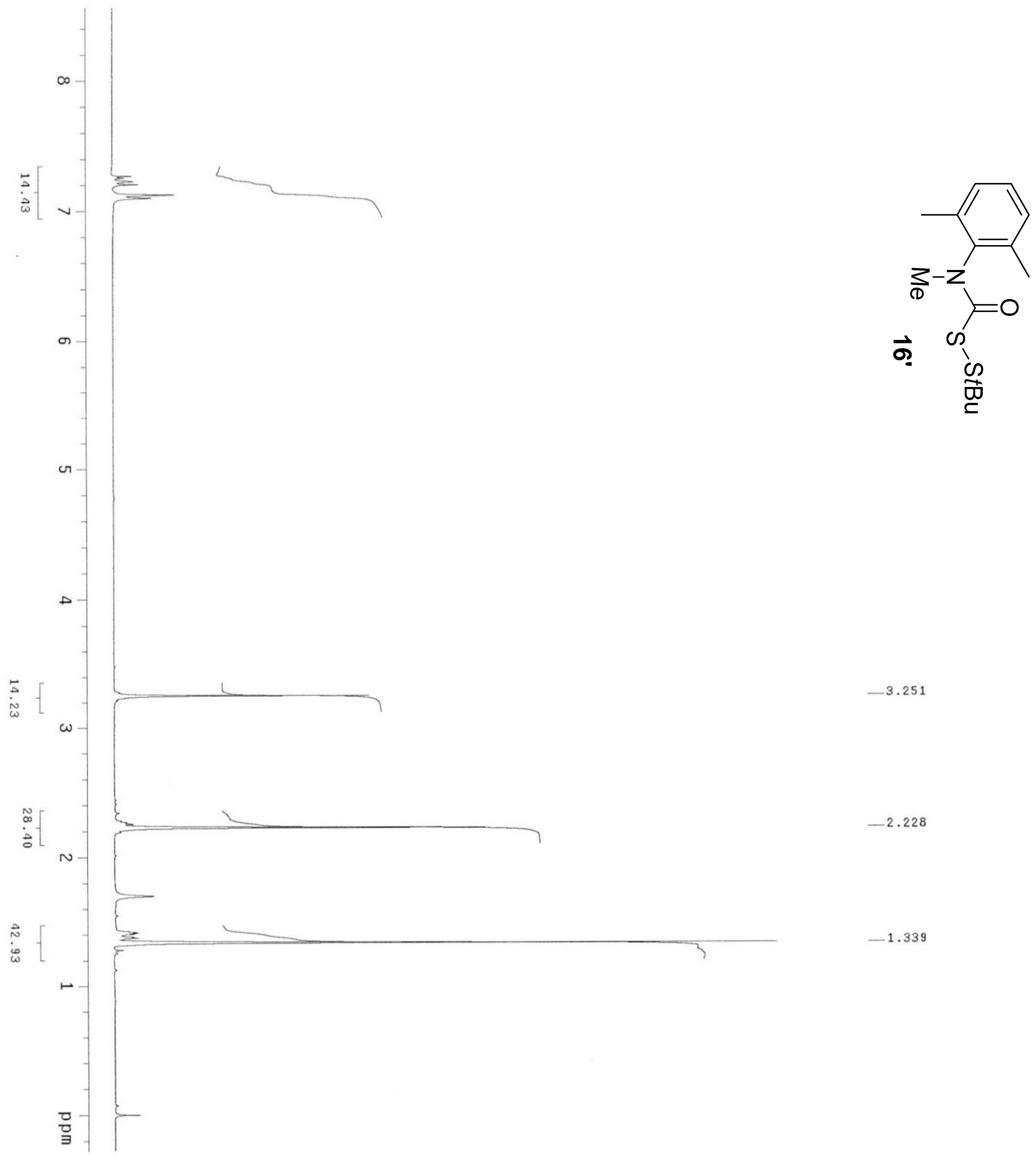

Figure S16. ^{13}C NMR of *tert*-butyl (*N*,2,6-trimethyl-*N*-phenylcarbamoyl)trisulfane (**16′**) (75 MHz)

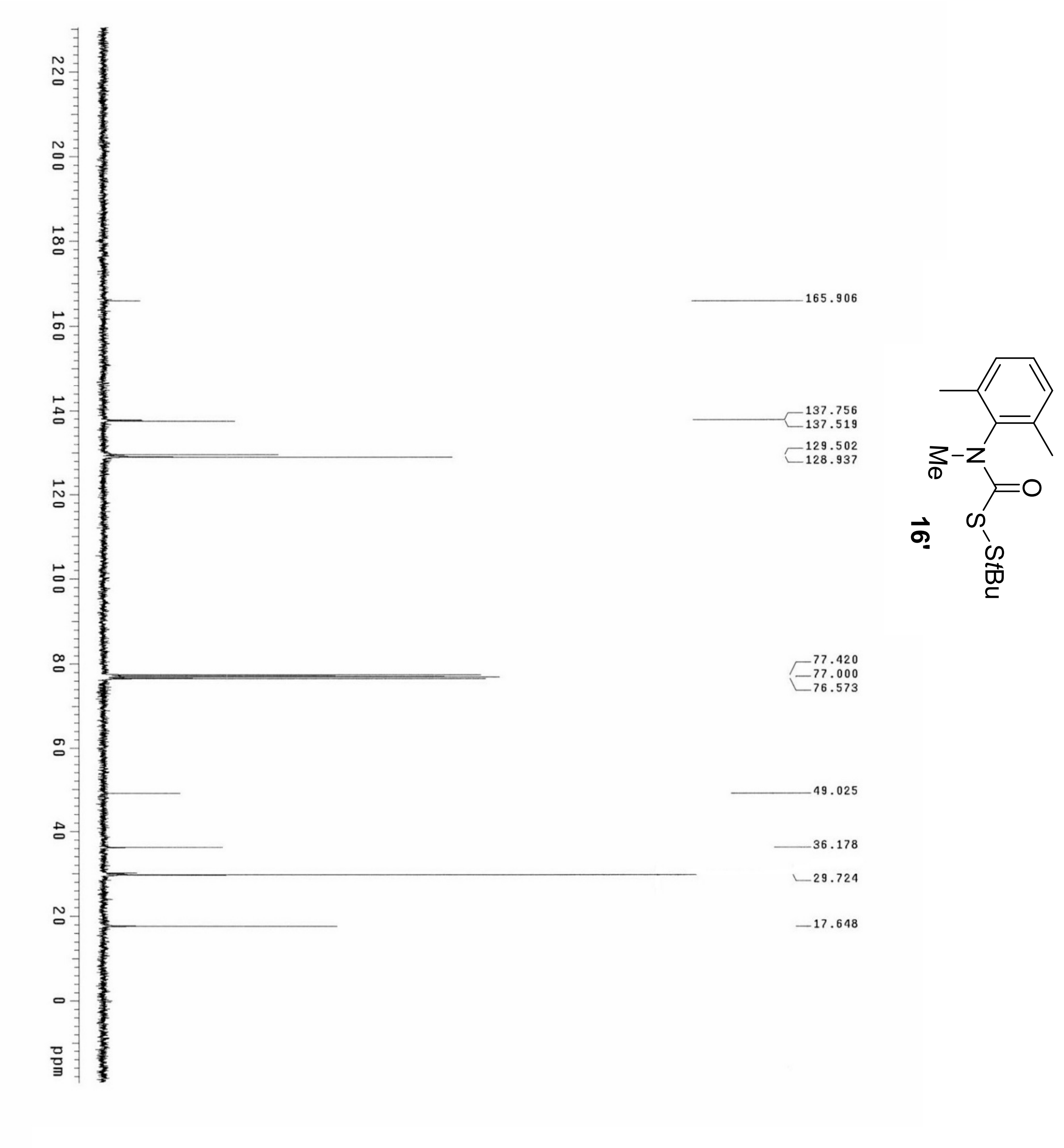

Figure S17. ¹H NMR of 4-methyl-2(3*H*)-benzo-1,2,4-dithiazinone (**3**) after treatment with excess triphenylphosphine, showing quantitative conversion to 3-methyl-2(3*H*)-benzothiazolone (**4**) (400 MHz)

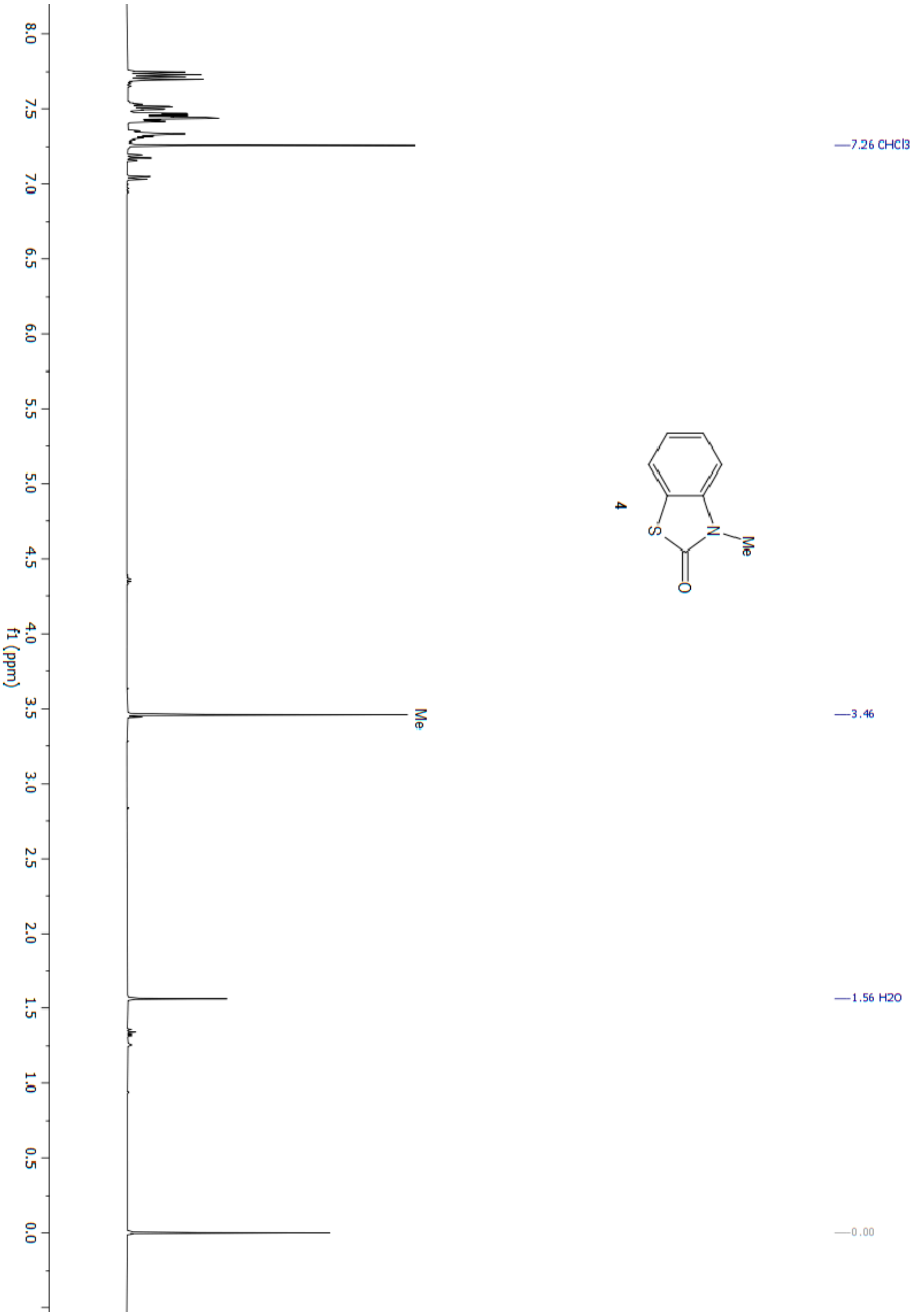

Figure S18. ^{13}C NMR of 4-methyl-2(3*H*)-benzo-1,2,4-dithiazinone (**3**) after treatment with excess triphenylphosphine, showing quantitative conversion to 3-methyl-2(3*H*)-benzothiazolone (**4**) (101 MHz)

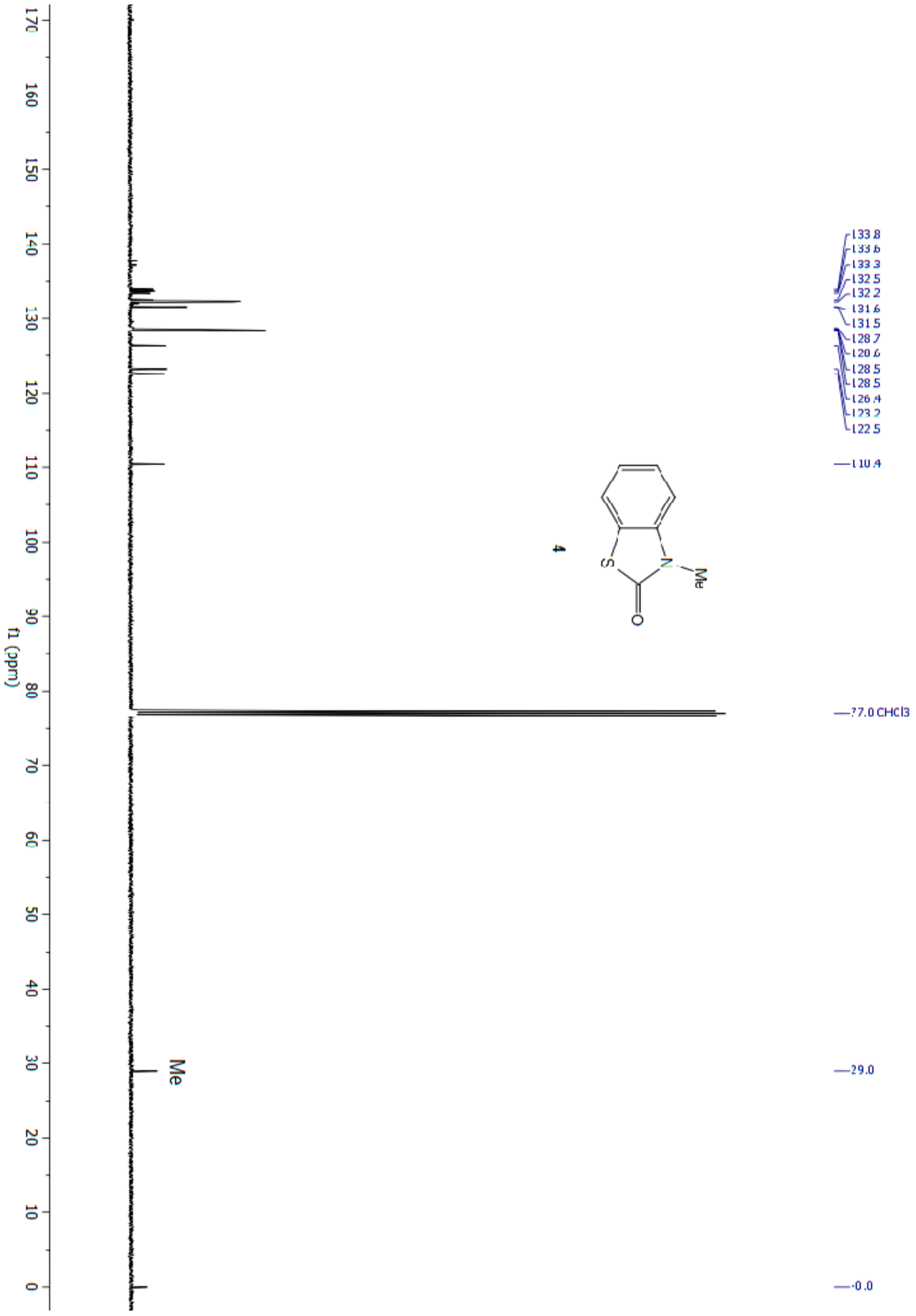

Figure S19. ^{31}P NMR of 4-methyl-2(3*H*)-benzo-1,2,4-dithiazinone (**3**) after treatment with excess triphenylphosphine, showing quantitative conversion to 3-methyl-2(3*H*)-benzothiazolone (**4**) (162 MHz)

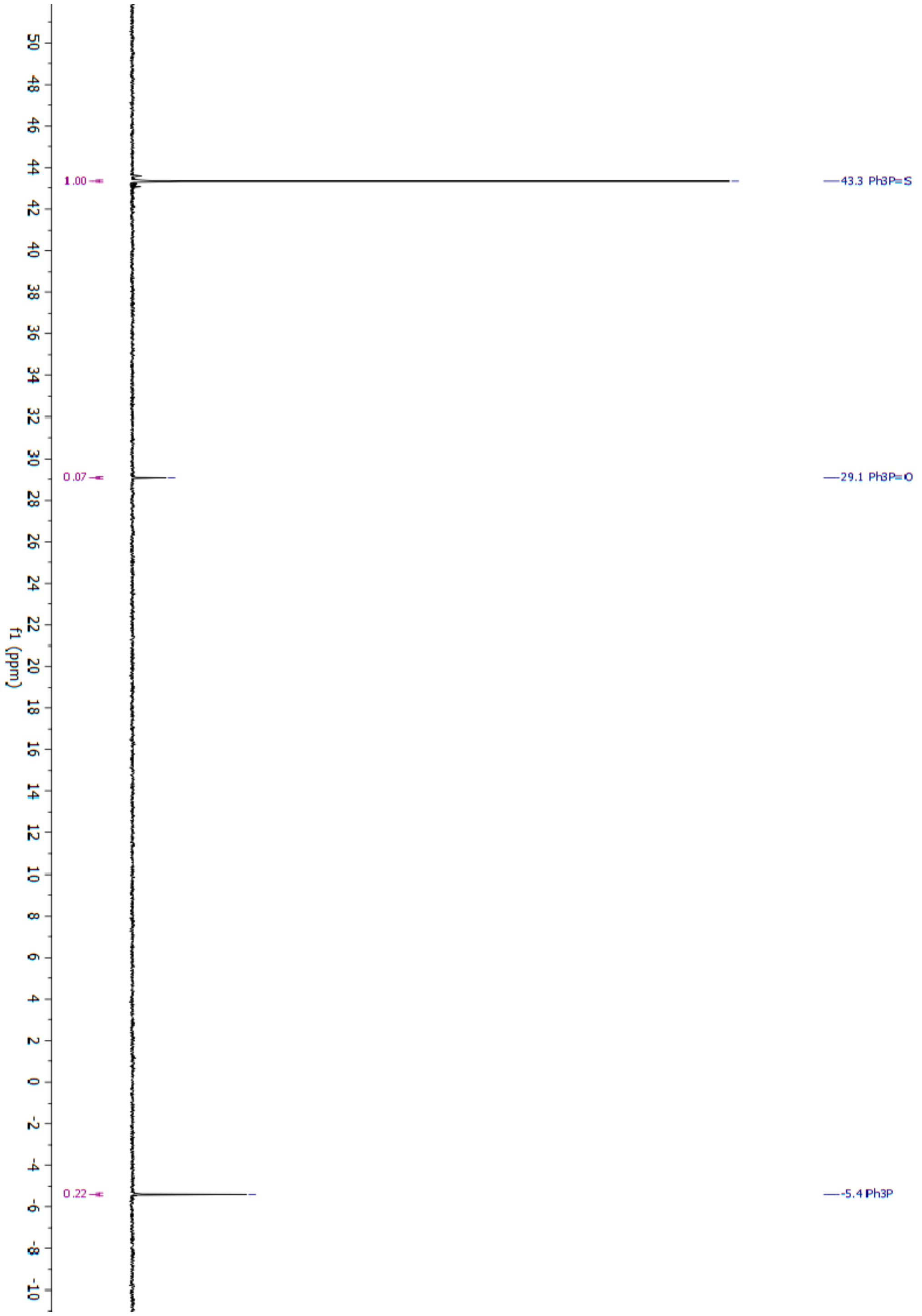

Figure S20. ^{1}H NMR of 4-methyl-2(*3H*)-benzo-1,2,4-dithiazinone (**3**) after treatment with limiting triphenylphosphine, showing quantitative conversion to triphenylphosphine sulfide (400 MHz)

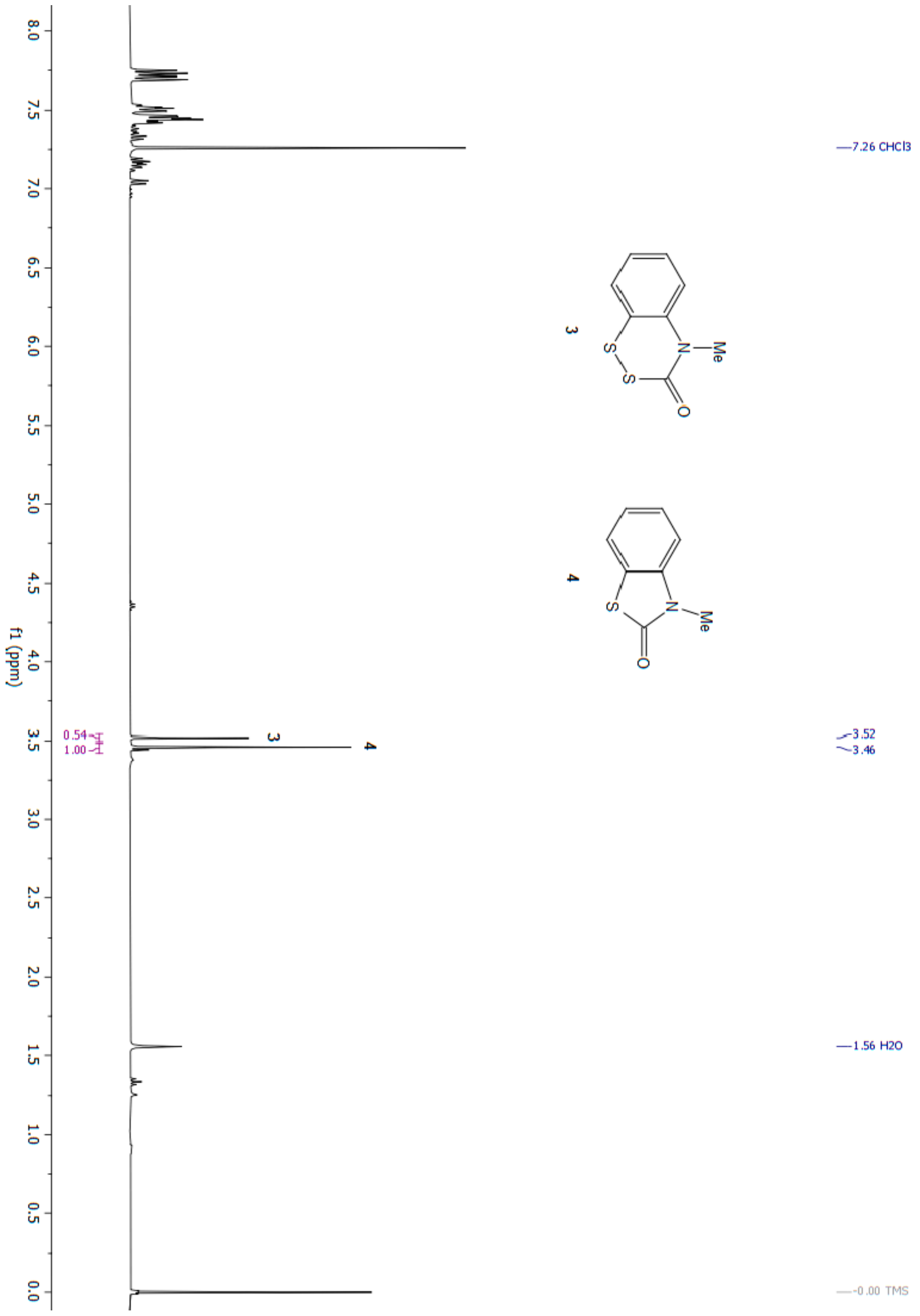

Figure S21. ^{13}C NMR of 4-methyl-2(*3H*)-benzo-1,2,4-dithiazinone (**3**) after treatment with limiting triphenylphosphine, showing quantitative conversion to triphenylphosphine sulfide (101 MHz)

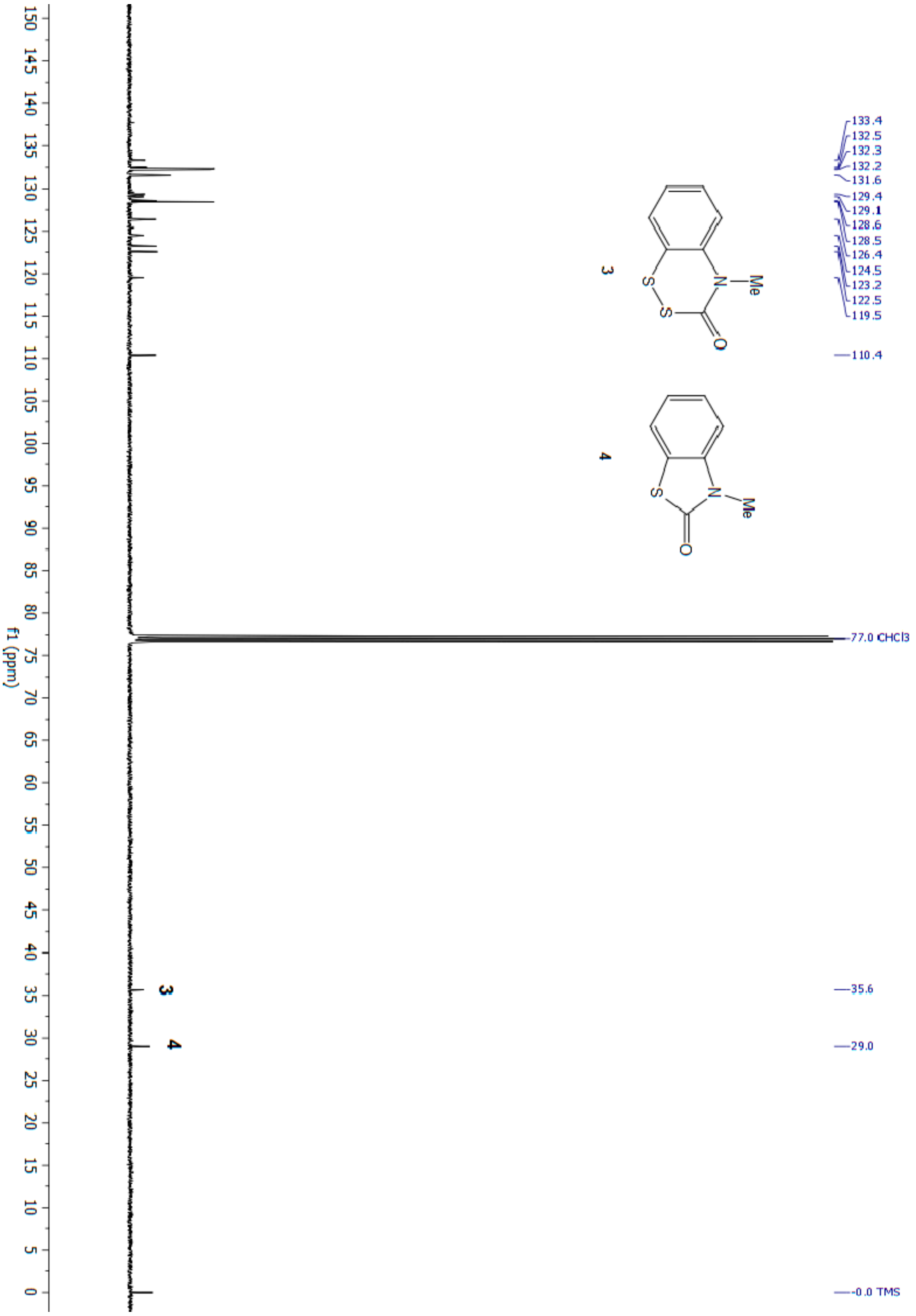

Figure S22. ^{31}P NMR of 4-methyl-2(*3H*)-benzo-1,2,4-dithiazinone (**3**) after treatment with limiting triphenylphosphine, showing quantitative conversion to triphenylphosphine sulfide (162 MHz)

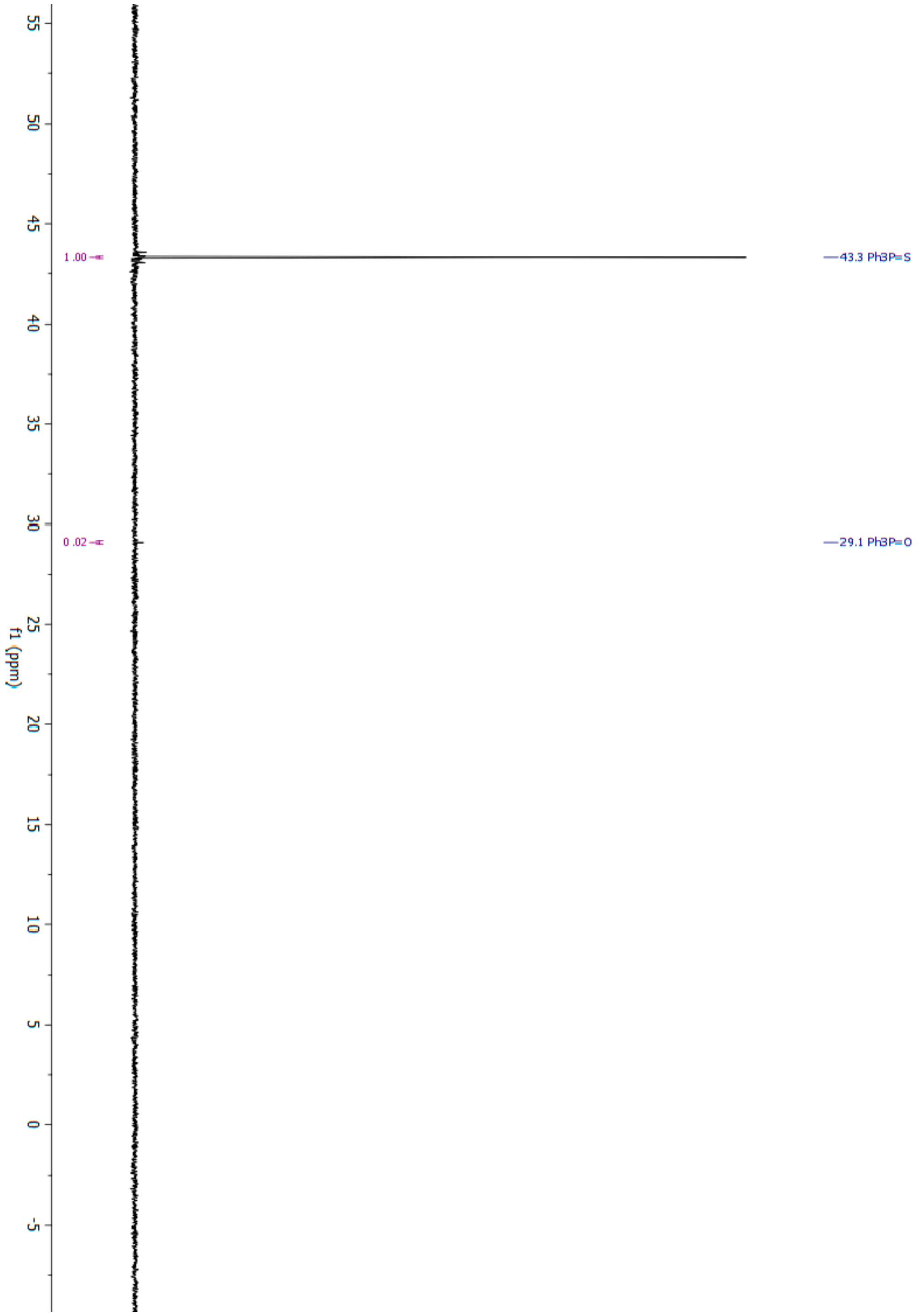

Supplement: Supplementary file 1 [file molecules-30-03892-s001.zip › molecules-3808997-supplementary.pdf]
